# Large temperature excursions have modest impacts on community composition in the high diversity gut microbiome of omnivorous American cockroaches (*Periplaneta americana*)

**DOI:** 10.1128/spectrum.00288-26

**Published:** 2026-06-01

**Authors:** Kevin C. Riedmuller, Josey E. Dyer, Elizabeth A. Ottesen

**Affiliations:** 1 Department of Microbiology, University of Georgia 189270, Athens, Georgia, USA; University of Arkansas Fayetteville, Fayetteville, Arkansas, USA

**Keywords:** Blattodea, gut microbiome, thermal stress, *Periplaneta americana*

## Abstract

Microbial residents of ectothermic hosts are exposed to variations in temperature that have the potential to impact their physiology and the host-microbe symbiotic relationship. In this experimental warming study, laboratory populations of American cockroaches (*Periplaneta americana*) were kept at a baseline low room temperature of 20–22°C or a high temperature of 30°C, for 2 weeks. We quantified bacterial load and performed high-throughput 16S rRNA gene sequencing to assess the hindgut microbiome’s response to a near 10°C shift in environmental temperature. We report modest impacts of temperature on cockroach gut microbiome composition. The high temperature treatment induced increases in the relative abundance of Proteobacteria and Euryarchaeota phyla, as well as the Lactobacillaceae and Enterococcaceae families. We also observed increased interindividual variability. There were no significant differences in the dominant Bacteroidota or Firmicutes phyla, and no significant losses or reductions in taxa, or bacterial load, respectively. This suggests that the gut community of American cockroaches is largely resilient to prolonged increases in temperature, and has implications for the cockroach to withstand the impacts of climate change.

**IMPORTANCE:**

Insects, as with most animals, often harbor microbial symbionts that play an essential role in host health and nutrition. As insects are ectotherms, these microbial symbionts are subject to the same temperature fluctuations as their hosts, potentially impacting host temperature responses. Here, we demonstrate that the American cockroach (*Periplaneta americana*) gut microbiome exhibits only modest changes following an ~10°C increase in environmental temperature. This contrasts with studies in other insects, whose microbiota were highly responsive to temperature variation. This work illustrates that the microbiota of insects may vary in their sensitivity to long-term temperature shifts, providing a more comprehensive understanding of potential variability in insect responses to climate change.

## INTRODUCTION

Insects are some of the most abundant multicellular organisms on Earth, with an estimated 5.5 million species (1). They are key drivers of ecological and biological processes ranging from nutrient cycling (2, 3) to disease dynamics (4, 5), and agricultural productivity (6, 7). As ectotherms, environmental temperature strongly governs their biological timing, physiology, distribution, and fitness (8). Consequently, changes in environmental temperature can have outsized and varied impacts on insects’ roles in these critical systems. For example, simulated warming in mesocosms containing *Formica subsericea*, an ant native to eastern North America, led to decreased decomposition rates and nitrogen availability (9). In contrast, a field study of *Formica manchu* nests on the southeastern Tibetan Plateau in China found that decomposition rates and nitrogen availability remained stable under simulated warming conditions (10). As the last 10 years have been the warmest on record and global surface temperatures are rising faster than any other period over the last 2,000 years (11, 12), understanding how insects are affected by warming climate has become increasingly important.

Insects have evolved a wide variety of behavioral and physiological thermoregulatory responses for survival that modulate metabolism, stress response, and growth and development (8). Studies frequently report faster growth and/or development rates in response to increased temperature, which is often accompanied by a decrease in body size (13–15). However, one aspect of insect adaptation to temperature that is less well-understood is the impact of temperature on the insect gut microbiome. The gut microbiome plays a critical role in host nutrition (16–18), development (19–21), and immunity (22–24). As symbionts of ectotherms, gut microbes (and the services they provide to their hosts) are subject to changes in environmental temperature alongside their hosts. Studies in three species of stink bugs have reported reduction or complete loss of gut bacterial symbionts in response to increased environmental temperature by as little as 2.5°C (25, 26). Similarly, a paired observational and experimental study in desert arboreal ants found that increased environmental temperature resulted in decreased bacterial loads as well as moderate shifts in microbial community composition. Notably, these changes in the gut microbiome were due to only a 1°C shift from baseline temperature in the experimental treatment (27). These data parallel studies in other ectotherms like corals, sponges, and mussels where increased temperature saw substantial shifts, reductions, or complete loss of symbiotic microbes (28–30). Overall, studies in a variety of invertebrates thus far tend to observe marked changes in both high and low diversity gut microbiomes and subsequent decreases in host fitness as a result of elevated temperatures ranging from ~1°C to 9°C from baseline temperatures.

Omnivorous cockroaches carry a high-diversity hindgut microbiome (31–33), which is believed to play an important role in facilitating host digestion of complex polysaccharides and other refractory dietary components (34, 35). The cockroach gut microbiome has been shown to play a vital role in shaping host development (36, 37), immune activity (38, 39), and behavior (40). It also shows evidence of long-term co-evolution with its host (41) and likely plays a key role in their effectiveness as decomposers and scavengers—functions that contribute significantly to global nutrient cycling due to their ubiquity across terrestrial ecosystems. For example, in central Amazonian inundation forests, at least eight different species of cockroaches were identified, one of which was estimated to consume up to 6% of annual leaf litter (42). Their effectiveness as decomposers and scavengers also contributes to pest species’ ability to infest virtually all human spaces (43). Pest species of cockroaches pose significant human health risks as they are a common cause of allergy and asthma (44, 45), and vectors for a variety of pathogens (46, 47). Moreover, they contribute to economic losses by driving up medical costs (48), destroying property and goods (43), and necessitating continual pest control (49). Given their ecological and economic importance, it is critical that we understand the effects of warming temperatures on the cockroach and its closely associated gut microbiome.

While little is known about the impact of temperature on the cockroach gut microbiome, studies of their close relatives, the termites (50), show marked temperature impacts. An experimental study in *Reticulitermes flavipes* comparing the gut microbiota at 15°C, 27°C (baseline), and 35°C found dramatic shifts in gut microbiome composition. Colder temperatures were associated with increased relative abundances of Proteobacteria, while heat stress was associated with decreased bacterial richness, loss of protist-associated bacteria, and increased relative abundances of Bacteroidota (51). In contrast, a field study in Janesville, Wisconsin, between July and December, observed only modest seasonal shifts in gut community composition of *R. flavipes*. Notably, the mean environmental air temperature during the collection period ranged from 22°C to −2°C, which was substantially lower than the warming treatment in the experimental study (52).

The aim of this work was to investigate the impacts of environmental temperature on the gut microbiome of the model insect *Periplaneta americana*. It has been observed that their thermal preference ranges from ~24°C to 33°C (53). However, in many cases, experimental insects are kept at a variety of temperatures within or below the thermal preference range (54–56), or at an unspecified room temperature (33, 57–59). While such experimental differences likely make little difference to the gut microbiota of endotherms such as mice, it poses a potential complicating factor in studies of microbial symbioses of ectotherms. As a result, we sought to examine the impact of temperature on the gut microbiota of omnivorous cockroaches. To do so, we employed bacterial load quantification and high-throughput 16S rRNA gene sequencing to assess the hindgut microbiome’s response to an increase in environmental temperature from a “low” laboratory room temperature of 20–22°C to 30°C, a “high” temperature used to speed reproduction and development (60). This work provides insight into how temperature can impact the gut microbiota of ectothermic hosts. This knowledge has implications for both organismal responses to climate shifts and experimental practice for laboratory investigation of ectotherm symbioses.

## MATERIALS AND METHODS

### Insects and experimental design

*P. americana* cockroach stock colonies have been maintained in captivity at room temperature (20–22°C) for over 15 years. Stock colonies live in 10-gallon glass aquarium tanks with wood chip bedding (PJ Murphy Forest Products SaniChips) and cardboard tubes for housing. They were provided dog chow (Purina One) and water (via cellulose sponge) *ad libitum*.

For temperature treatments, 20–24 mixed-sex, adult cockroaches were selected from stock colonies, divided into two plastic terrariums, and maintained under stock colony conditions at room temperature (20–22°C) or 30°C for 14 days. Cohort and treatment sample sizes are located in Table S1. Dog chow with any visible mold growth was removed daily. The experiment was performed in duplicate. Cohorts 1 and 2 treatments were completed in April of 2022; cohort 2 treatments were initiated 1 week after cohort 1 began. We also include data from a pilot experiment conducted in November 2020 (cohort 3). The baseline room temperature for this cohort was slightly warmer, ranging from 22°C to 25°C.

### Sample collection and DNA extraction

Cockroaches were placed on ice in sterile petri dishes until torpid (~5 min). The entire gut (foregut to rectum) was excised with a scalpel and forceps; any visible fat body and exoskeleton was removed. The gut tract was then moved to a sterile aluminum dish, placed over dry ice, and the hindgut was resected. Hindguts were transferred to 500 µL of sterile 1× phosphate-buffered saline and stored at −20°C.

Hindguts were thawed and homogenized individually with a sterile microcentrifuge pestle. DNA was extracted from a 200 µL aliquot of each sample homogenate using the E.Z.N.A Bacterial DNA Kit (Omega Bio-tek, Norcross, GA, USA) per the manufacturer’s protocol with the following modifications: 30 min lysozyme incubation, optional glass bead beating step included, and RNase A step excluded. Sample DNA was eluted in 50 µL of elution buffer. DNA concentrations and *A*_260/280_ values were measured with a BioTek Synergy HTX Multimode plate reader (Agilent, Santa Clara, CA, USA). Sample DNA was stored at −20°C and concentrations ranged from 30 to 300 ng/µL (Table S2).

### Bacterial load quantification

Total number of bacteria per sample was assessed via qPCR of the 16S rRNA gene as described in Nadkarni et al. (61). qPCR was performed in triplicate, 10 µL reactions using a QuantStudio 6 flex instrument and QuantStudio Real-Time PCR Software (v1.3) (Applied Biosystems, Waltham, MA, USA). Reactions consisted of 1× TaqMan Multiplex Master Mix (Applied Biosystems), 0.3 µM forward primer 5′-TCCTACGGGAGGCAGCAGT-3′ (Tm, 59.4°C), 0.3 µM reverse primer 5′-GGACTACCAGGGTATCTAATCCTGTT-3′ (Tm, 58.1°C), 0.25 µM hydrolysis probe (6-FAM)−5′-CGTATTACCGCGGCTGCTGGCAC-3′-(TAMRA) (Tm, 69.9°C), and 1 µL sample DNA (see Table S2 for DNA quantities) under the following conditions: 95°C for 20 s, followed by 40 cycles of 95°C for 1 s and 60°C for 60 s. A standard curve was generated with a 10-fold dilution series of gBlocks Gene Fragment of the *Frigididesulfovibrio cuneatus* 16S gene (GenBank: X99503.1, positions 330–798) with a linear dynamic range of 10–10^8^ copies. The slope, *y*-intercept, and *r*^2^ of the standard curve was −3.529, 41.67, and 0.994, respectively. Amplification efficiency was 92.04%.

qPCR of a *Blattabacterium* MIP/aquaporin family protein coding sequence was performed to account for the cockroach endosymbiont’s contribution to bacterial load. Triplicate, 10 µL reactions consisted of 1× TaqMan Environmental Master Mix 2.0 (Applied Biosystems), 0.1 µM forward primer 5′-CAGCTAATGCCCATCCCATAG-3′ (Tm, 55.4°C), 0.1 µM reverse primer 5′-GGAAATGGAGTGGTGGCTAAT-3′ (Tm, 55.0°C), 0.1 µM hydrolysis probe (6-FAM)−5′-TTGTGACCCATCCACCATCTCCAT-3′-(MGB-NFQ) (Tm, 63.7°C), and 1 µL sample DNA under the following conditions: 95°C for 10 min followed by 40 cycles of 95°C for 15 s and 60°C for 60 s. A standard curve was generated with a 10-fold dilution series of gBlocks Gene Fragment (CAGCTAATGCCCATCCCATAGTGATTGTGACCCATCCACCATCTCCATGTCCTTTTGTTTTAGACAATAGAACATTAGCCACCACTCCATTTCC) with a linear dynamic range of 10–10^8^ copies. The slope, *y*-intercept, and *r*^2^ of the standard curve was −3.526, 38.97, and 0.993, respectively. Amplification efficiency was 92.15%.

*C*_q_ values for both assays were auto selected by the QuantStudio Real-Time PCR Software (v1.3). All primers and gBlocks were synthesized by Integrated DNA Technologies with standard desalting purification. Hydrolysis probes were synthesized by Applied Biosystems. Primer set specificity was validated via endpoint PCR and agarose gel electrophoresis.

Hindgut bacterial load L was calculated as follows:


L=((s−b)/c)×t

where s= 16S copy number, b= *Blattabacterium* MIP/aquaporin family protein copy number, c= DNA quantity in qPCR reaction (ng), and t= total sample DNA (ng). Reported 16S and *Blattabacterium* MIP/aquaporin family protein copy numbers are averages from triplicate qPCR reactions. Bacterial load boxplots were generated with the Tidyverse package (v2.0.0) (62).

### Library preparation and sequencing

The V4 region of the 16S rRNA gene from each hindgut sample was amplified using a two-step PCR protocol as described in Tinker et al. (63). Initial amplification of the V4 region was performed with a 10 µL PCR reaction that consisted of 1× Q5 hot start reaction buffer, 0.02 U/µL Q5 hot start high-fidelity DNA polymerase (New England Biolabs, Ipswitch, MA, USA), 0.5 µM 515F primer 5′-GTGCCAGCMGCCGCGGTAA-3′, 0.5 µM 806R primer 5′- GGACTACHVGGGTWTCTAAT-3′, 200 µM deoxynucleotide triphosphates (dNTPs), and 0.8 ng/µL sample DNA under the following conditions: 98°C for 30 s, followed by 15 cycles of 98°C for 10 s, 52°C for 30 s, and 72°C for 30 s, followed by a final extension step at 72°C for 2 min. The product of reaction 1 was reamplified in a subsequent 30 µL PCR reaction, containing 1× Q5 hot start reaction buffer, 0.02 U/µL Q5 hot start high-fidelity DNA polymerase, 0.5 µM 515F barcoded primer, 0.5 µM 806R barcoded primer, 200 µM dNTPs, and 9 µL of reaction 1 product under the following conditions: 98°C for 30 s, followed by four cycles of 98°C for 10 s, 52°C for 30 s, and 72°C for 30 s, followed by six cycles of 98°C for 10 s and 72°C for 1 min, followed by a final extension step at 72°C for 2 min. All primers were synthesized by Integrated DNA Technologies with standard desalting purification (Coralville, IA, USA).

PCR products were verified by agarose gel electrophoresis and purified with the E.Z.N.A Cycle Pure Kit (Omega Bio-tek) per the manufacturer’s protocol and eluted in 30 µL elution buffer. Purified amplicon concentrations and *A*_260/280_ values were measured with a BioTek Synergy HTX Multimode plate reader. Amplicons were normalized and pooled to a final concentration of 10 ng/µL. The quality of the prepared amplicon library was assessed with the Agilent 2100 Bioanalyzer DNA-HS assay (Agilent) before submission to the Georgia Genomics and Bioinformatics Core for paired-end 250 bp sequencing on an Illumina MiSeq platform (Illumina Inc., San Diego, CA, USA).

### Sequence processing

Demultiplexed, paired-end fastq files were analyzed with the standard DADA2 pipeline (v1.38.0) (64). Forward reads were truncated at 240 bp and reverse reads were truncated at 160 bp. Samples were pooled during the sample inference step to increase sensitivity to sequence variants present at low abundances in multiple samples. Taxonomy was assigned to amplicon sequence variants (ASVs) with the assignTaxonomy() function of the DADA2 package (default minimum bootstrap confidence of 50) and the Silva reference database (v138.1) (65). assignTaxonomy() output was passed to the addSpecies() function, adding species level assignments to ASVs provided there was a 100% match to a reference strain. Reads corresponding to *Blattabacterium* (cockroach endosymbiont located in the fat body), chloroplasts, and mitochondria were removed. Samples were processed in batches according to sequencing run to account for run-specific sequencing errors (cohort 3 processed separately from cohorts 1 and 2). The batches were subsequently merged prior to the chimera removal step of the pipeline. All downstream analyses of sequencing and bacterial load data were performed using R Statistical Software (v4.5.2) (66) and Geneious Prime 2026.0.2 (https://www.geneious.com).

### Alpha and beta diversity

All libraries were resampled to the lowest observed library depth with the Vegan package (v2.7.3) (67) for alpha and beta diversity metrics. Alpha diversity was assessed by calculating the Shannon diversity index, richness (number of observed taxa), and Pielou’s evenness (Shannon diversity index/log(richness)) (67). Alpha diversity was visualized with violin plots via the Tidyverse package (v2.0.0) (62). Beta diversity was assessed by calculating weighted and unweighted (incidence-based, equivalent to Sørensen index) (68) Bray-Curtis dissimilarities (67). Beta diversity was visualized with nonmetric multidimensional scaling ordinations and violin plots via the Tidyverse and Vegan packages (62, 67).

The ConQuR batch correction package (v2.0) (69) was implemented to account for cohort variation prior to alpha and beta diversity comparisons across temperature treatments. The ASV raw counts table was passed to the ConQuR_libsize() function. Cohort 1 was selected as the batch reference. Temperature and sex were included as covariates. The batch-corrected ASV count table was handled as previously described for alpha and beta diversity metrics. Correction for cohort was confirmed by PERMANOVA on Bray-Curtis dissimilarities (weighted *P* = 0.10, unweighted *P* = 0.17).

### Differential abundance analysis

DESeq2 (v1.50.2) (70) was implemented for ASV-level differential abundance analysis to identify temperature-responsive taxa. The ASV raw counts table was passed to the DESeq() function with default parameters and “design = ~cohort + sex + temperature,” testing for the effect of temperature while controlling for sex and cohort variation. ASVs with an adjusted *P* value < 0.05 were considered significant (Table S3). A phylogenetic tree of differentially abundant ASVs present in the 12 most abundant families (maximum relative abundance >10%) was generated in Geneious Prime. Sequences were aligned with Clustal Omega (v1.2.3) (71) and the phylogenetic tree was built using FastTree (v2.1.11) (72, 73). The *Desulfovibrionaceae* clade was used as an outgroup for rooting, and tree visualization was performed in R with the Tidyverse and ggtree (v4.0.5) (74) packages. A heatmap of differentially abundant ASVs present in the 12 most abundant families was generated with the ComplexHeatmap package (v2.26.1) (75, 76).

### Statistical analysis

Wilcoxon rank-sum tests, a non-parametric test comparing the distribution of independent groups, were used for two-group comparisons. Kruskal-Wallis tests, an extension of the Wilcoxon rank-sum test, were used for the overall comparison of three or more groups (non-parametric ANOVA analog). Significant Kruskal-Wallis tests were followed up with post-hoc Dunn’s test with Bonferroni adjustment to determine which groups’ distributions were significantly different (FSA v0.10.1) (77). Permutational multivariate analysis of variation (PERMANOVA) was applied to Bray-Curtis dissimilarity matrices to determine whether groups differed in overall community composition using the adonis2() function of the Vegan package (67).

## RESULTS

### Hindgut bacterial load is stable across temperature treatments

**Fig 1:**
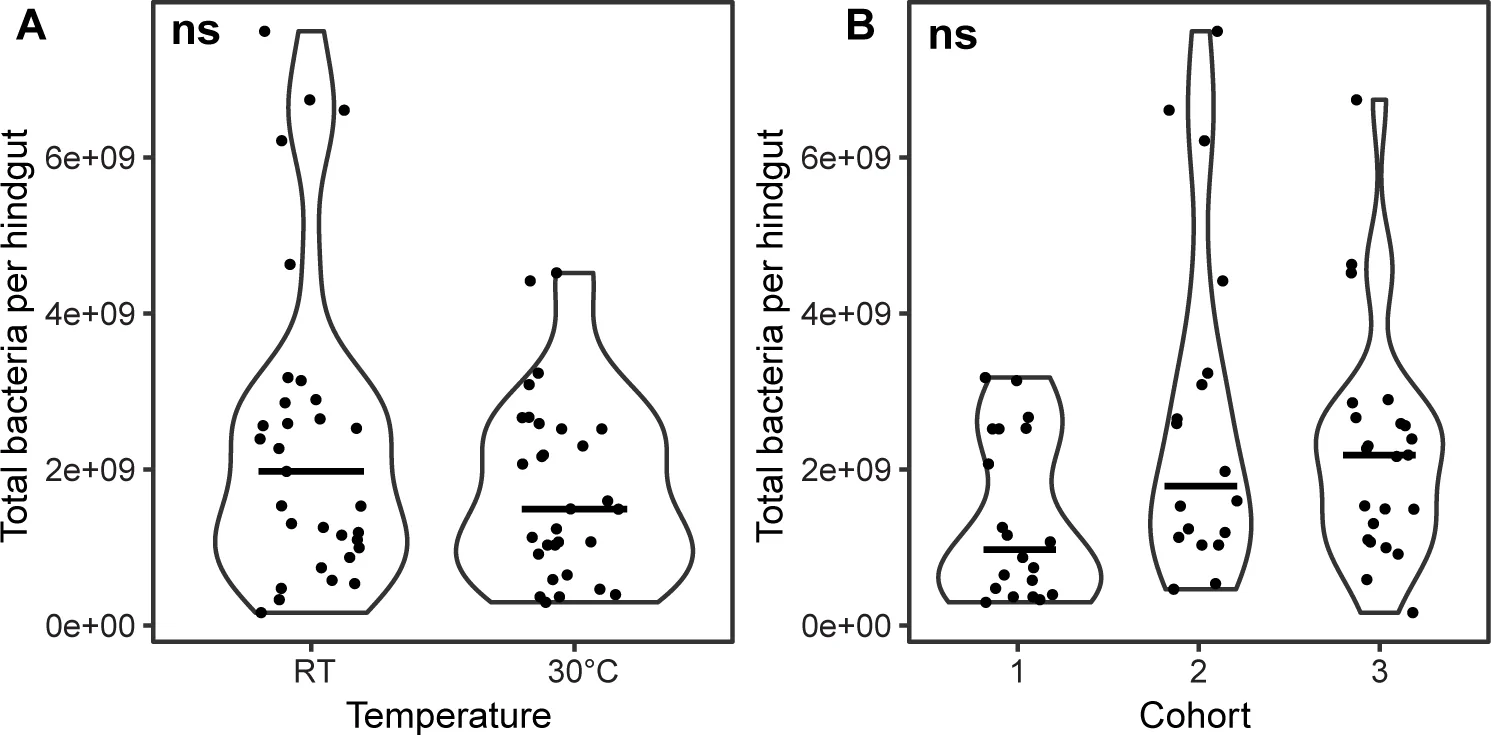
Comparison of hindgut bacterial load across temperature treatments and cohorts. Violin plots of hindgut bacterial load across (**A**) temperature and (**B**) cohort, where bars represent the median and points represent individual samples. Wilcoxon rank-sum test was used to compare across temperature treatments. Kruskal-Wallis and post-hoc Dunn’s test with Bonferroni adjustment was used to compare across cohorts. *Blattabacterium* counts subtracted from total 16S rRNA copy number. Bacterial load normalized by total sample DNA. ns, no significance; RT, room temperature.

To assess the effect of temperature on hindgut bacterial load, we performed qPCR of the 16S rRNA gene with a universal primer/probe set as described in Nadkarni et al. (61). To account for 16S rRNA copy contribution from the cockroaches’ *Blattabacterium* endosymbiont, we also performed qPCR of a *Blattabacterium* gene in the MIP/aquaporin family. A Wilcoxon rank-sum test found no significant difference in hindgut bacterial load across temperature treatments (*P* = 0.26). A Kruskal-Wallis test found a significant difference in hindgut bacterial load among cohorts (*P* = 0.04). However, Dunn’s test did not indicate significant differences between cohorts: cohorts 1 and 2 (*P* = 0.10), cohorts 1 and 3 (*P* = 1.00), and cohorts 2 and 3 (*P* = 0.07) (Fig. 1).

### Hindgut microbial community composition

A total of 4,526,160 rRNA gene sequences were obtained and 2,580,005 passed filtering parameters, resulting in an average of 42,295 reads per sample. One sample was removed prior to downstream analysis due to low sequencing depth (Table S1). 2,923 unique ASVs were identified across all samples.

**Fig 2:**
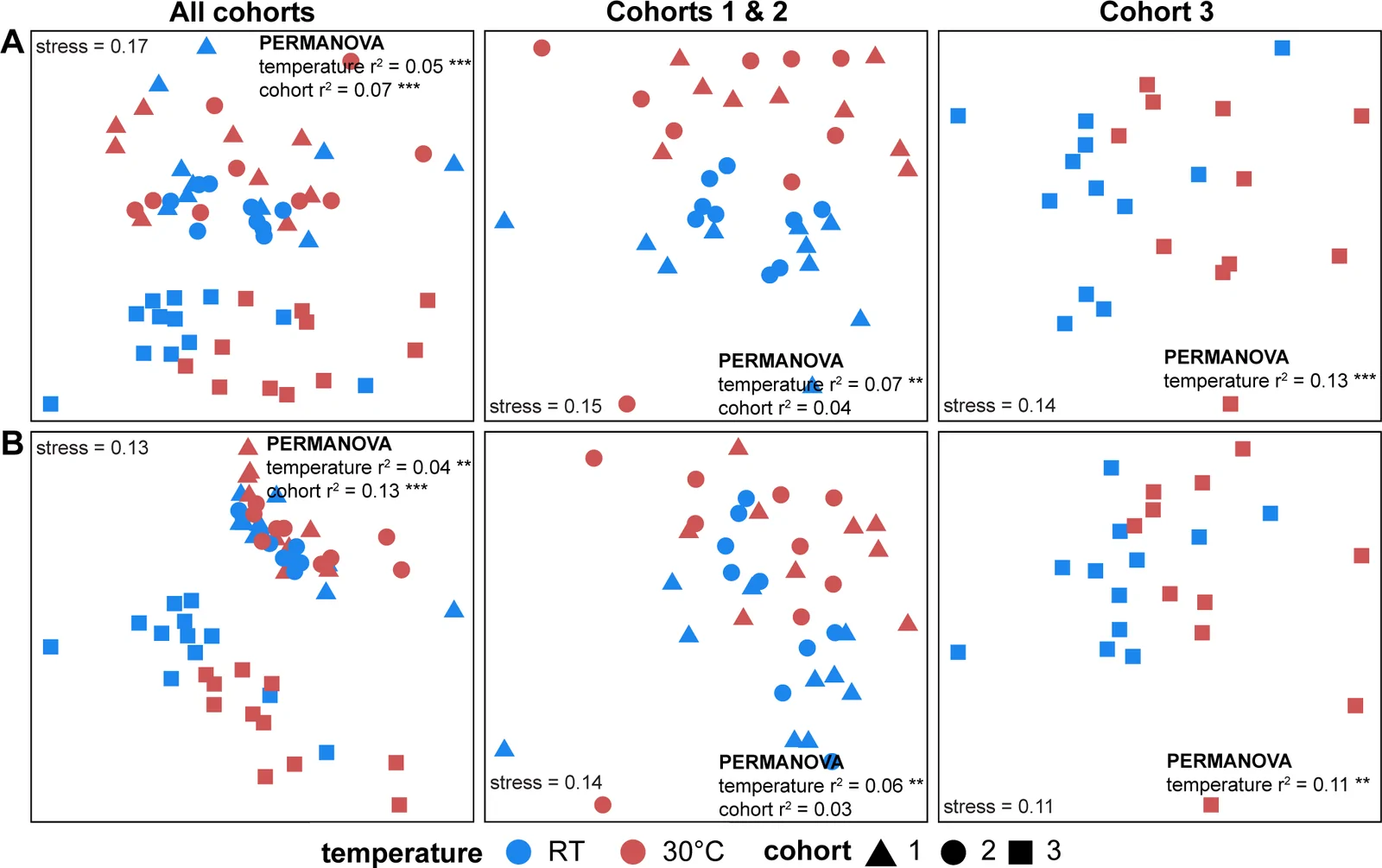
ASV level comparison of Bray-Curtis dissimilarities across cohorts. Nonmetric multidimensional scaling (NMDS) of (**A**) weighted and (**B**) unweighted Bray-Curtis dissimilarities at the ASV level without batch correction. NMDS stress was calculated with the metaMDS() function from the Vegan package. PERMANOVA was used to calculate *r*^2^ and *P* values for temperature and cohort. All libraries were resampled to a depth of 10,097 reads. RT = room temperature; **P < 0.01, ***P < 0.001; ns, no significance.

Given the large time gap between experiments, we first examined cohort impacts on community composition. Notably, nonmetric multidimensional scaling (NDMS) of weighted and unweighted Bray-Curtis dissimilarities showed that cohort 3 samples tended to cluster separately from other cohorts and permutational multivariate analysis of variance (PERMANOVA) found a significant effect for cohort on community composition at the ASV level (weighted: *P* = 0.001, *r*^2^ = 0.07; unweighted: *P* = 0.001, *r*^2^ = 0.13) (Fig. 2). Dunn’s test indicated significantly greater between-cohort dissimilarity for all comparisons involving cohort 3 compared to the between-cohort dissimilarity of cohorts 1 and 2 by weighted and unweighted metrics at the ASV level (*P* < 0.001) (Fig. S1). This suggests that some observed taxa in cohort 3 differ from the observed taxa in cohorts 1 and 2. This trend aligns temporally with each experiment, where cohort 1 and 2 treatments were carried out within a week of each other while cohort 3 was carried out over one year earlier. This indicates minor, detectable drift in taxonomic composition of our stock colonies.

To investigate whether cohort effects were obscuring temperature responses, we examined temperature effects on gut microbiome composition for all three cohorts together, cohorts 1 and 2 (combined due to their more similar community composition), and cohort 3 alone (Fig. 2). Samples separated more clearly by temperature, although the effect size of temperature calculated by PERMANOVA was modest (all cohorts: weighted *P* = 0.001, *r*^2^ = 0.05 unweighted *P* = 0.01, *r*^2^ = 0.04; cohorts 1 and 2: weighted *P* = 0.002, *r*^2^ = 0.07; unweighted *P* = 0.008, *r*^2^ = 0.06; cohort 3: weighted *P* = 0.001, *r*^2^ = 0.13; unweighted *P* = 0.002, *r*^2^ = 0.11). To better account for cohort variation, we applied a batch correction algorithm from the ConQuR package to our ASV count table prior to any further community-wide alpha and beta diversity analyses (69).

**Fig 3:**
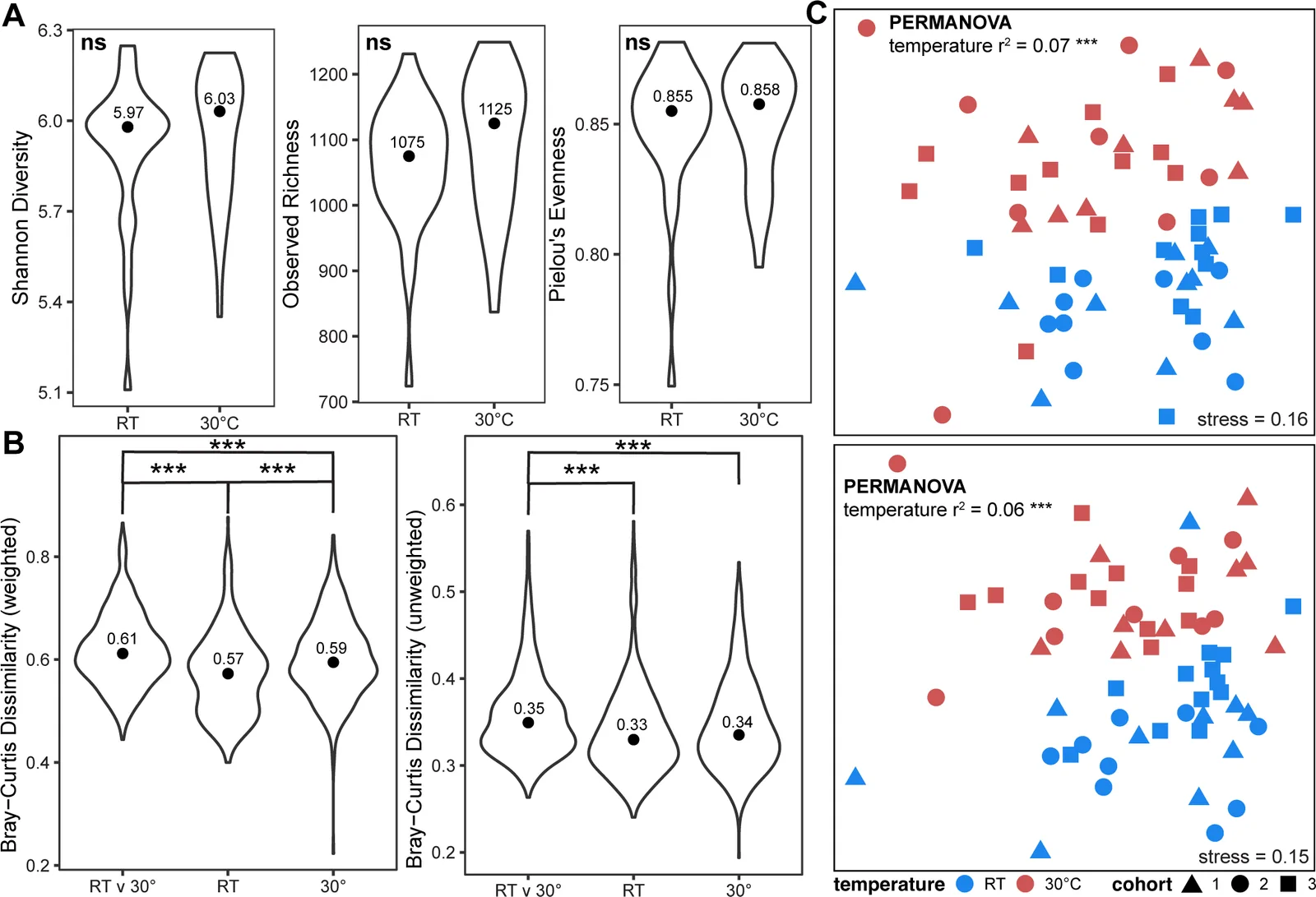
ASV level comparison of hindgut microbial community composition across temperature treatments. (**A**) Violin plots of alpha diversity measurements (Shannon diversity, observed richness, and Pielou’s evenness), where points represent the medians. (**B**) Violin plots of weighted (left) and unweighted (right) Bray-Curtis dissimilarities within and between temperature treatments where points represent the medians. Wilcoxon rank-sum tests were used to compare alpha diversity measures. Kruskal-Wallis and post-hoc Dunn’s test with Bonferroni adjustment were used to compare Bray-Curtis dissimilarities. (**C**) Nonmetric multidimensional scaling (NMDS) of weighted (top) and unweighted (bottom) Bray-Curtis dissimilarities. NMDS stress was calculated with the metaMDS() function from the Vegan package. PERMANOVA was used to calculate *r*^2^ and *P* values. All libraries were batch corrected and resampled to a depth of 10,098 reads. ns, no significance; RT, room temperature; ***P < 0.001.

Overall gut microbiome composition exhibited modest changes in response to temperature. Wilcoxon rank-sum tests found no significant differences in the Shannon diversity, richness (total number of ASVs), or Pielou’s evenness after batch correction at the ASV level (Shannon: *P* = 0.33; richness: *P* = 0.14; Pielou’s: *P* = 0.69) (Fig. 3A). Richness was the only measurement found to be significantly greater in the 30°C treatment at the family level with a median difference of 2 (Shannon: *P* = 0.35; richness: *P* = 0.002; Pielou’s: *P* = 0.31) (Fig. S2A). Beta diversity measures suggest modest but significant impacts of temperature on gut microbiome community composition. Dunn’s test indicated that between-temperature treatment dissimilarities were significantly greater than within-temperature treatment dissimilarities at the ASV level (*P* < 0.001) (Fig. 3B). Temperature was also observed to have impacts on beta dispersion, with the 30°C treatment having higher within-group dissimilarity (weighted) than the room temperature treatment (Dunn’s test: ASV *P* < 0.001; family *P* = 0.006) (Fig. 3B; Fig. S2B). An NMDS of weighted and unweighted Bray-Curtis dissimilarities showed clear separation of samples by temperature, and PERMANOVA found a significant effect at the ASV level (weighted: *P* = 0.001, *r*^2^ = 0.07; unweighted: *P* = 0.001, *r*^2^ = 0.06) (Fig. 3C). PERMANOVA also found a significant effect at the family level (weighted: *P* = 0.002, *r*^2^ = 0.04; unweighted: *P* = 0.003, *r*^2^ = 0.05) (Fig. S2C). However, samples show limited separation by temperature, and the NMDS stress (≥0.20) suggests that the ordination does not adequately capture dissimilarities in two-dimensional space. This likely reflects low overall variation in community composition and suggests greater compositional stability at the family level.

We observed sex-based differences in community composition based on weighted Bray-Curtis dissimilarities (PERMANOVA: *P* = 0.001, *r*^2^ = 0.15) (Fig. S3). However, the patterns described above remained the same when examining the effects of temperature on males and females separately (Fig. S4 and S5).

### Taxa exhibiting significant responses to temperature differences

**Fig 4:**
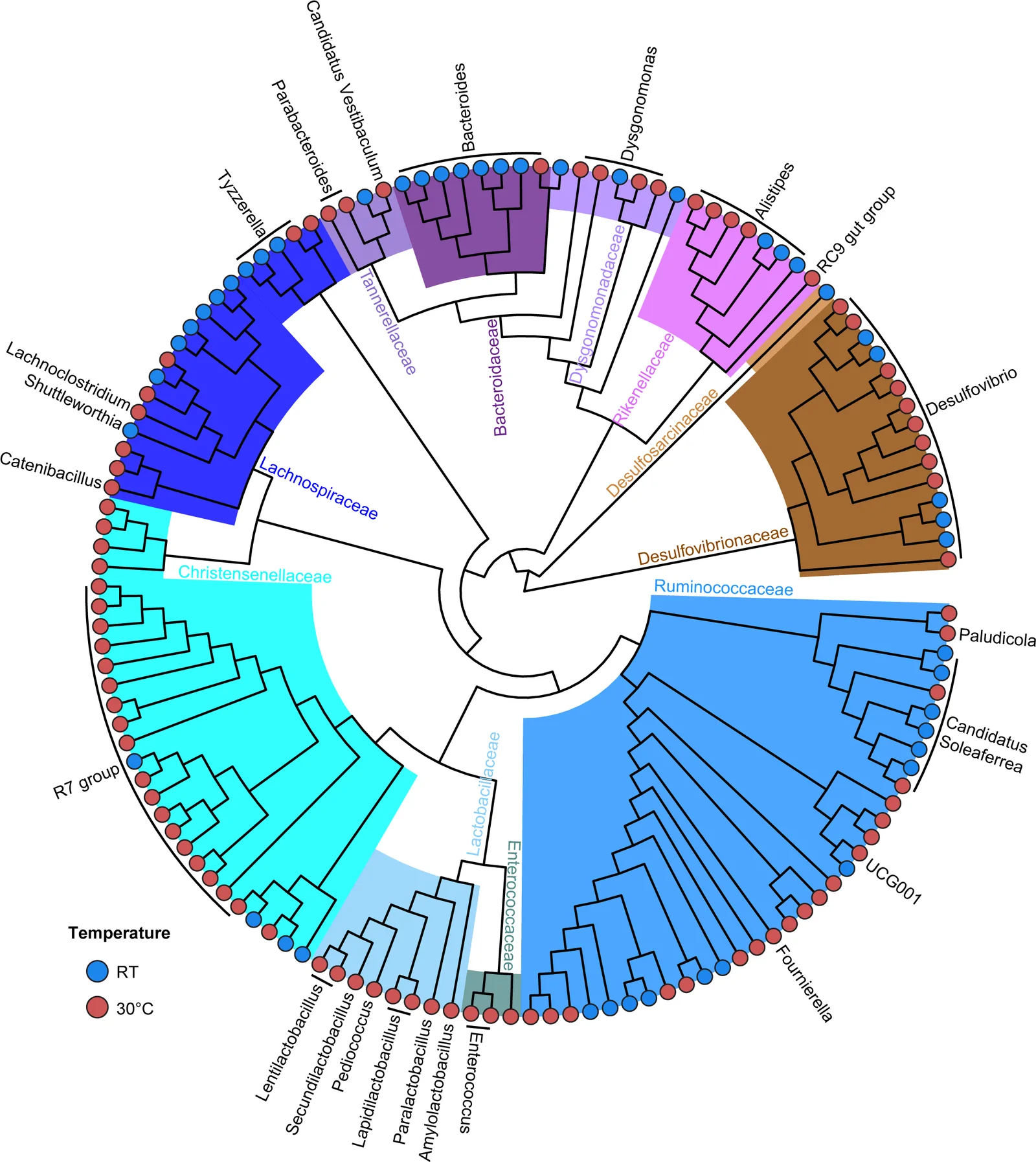
Phylogenetic tree of differentially abundant ASVs across temperature treatments. FastTree depicting differentially abundant ASVs in the most abundant families (maximum relative abundance >10%). Highlights represent family level classifications. Leaf tip labels represent genus-level classifications. ASVs without a leaf tip label were not classifiable at the genus level. Leaf tip colors depict which treatment ASVs were enriched in. Branch lengths not shown. RT, room temperature.

All ASVs present in more than 50% of the room temperature samples were also present in the 30°C samples, and vice versa, suggesting that temperature differences in community composition were primarily the result of changes in ASV relative abundance. We performed differential abundance analysis with DESeq2 to further explore ASV level responses to environmental temperature. Accounting for sex and cohort variation, 245 ASVs were identified as significantly differentially abundant (*P* < 0.05) across temperature treatments (Table S3). Of those 245 ASVs, 132 belonged to the most abundant families (maximum relative abundance >10%) in our samples (Fig. 4; Fig. S6). Given their low abundance and zero-inflated nature, the biological pertinence of any individual ASV identified as significant should be interpreted with caution (Fig. S7). Instead, we emphasize broader community-level trends. Ruminococcaceae, Lachnospiraceae, Rikenellaceae, and Dysgonomonadaceae families had a comparable number of ASVs enriched in both temperature treatments. This may be indicative of ASV-level switching among functionally redundant taxa within these families. However, we did not see this trend for the remaining families. All but one Bacteroidaceae ASV was enriched in the room temperature treatment, and all Lactobacillaceae and Enterococcaceae ASVs were enriched in the 30°C treatment. We found that most differentially abundant ASVs in the Tannerellaceae, Christensenellaceae, and Desulfovibrionaceae families were enriched in the 30°C treatment (Fig. 4; Fig. S6).

**Fig 5:**
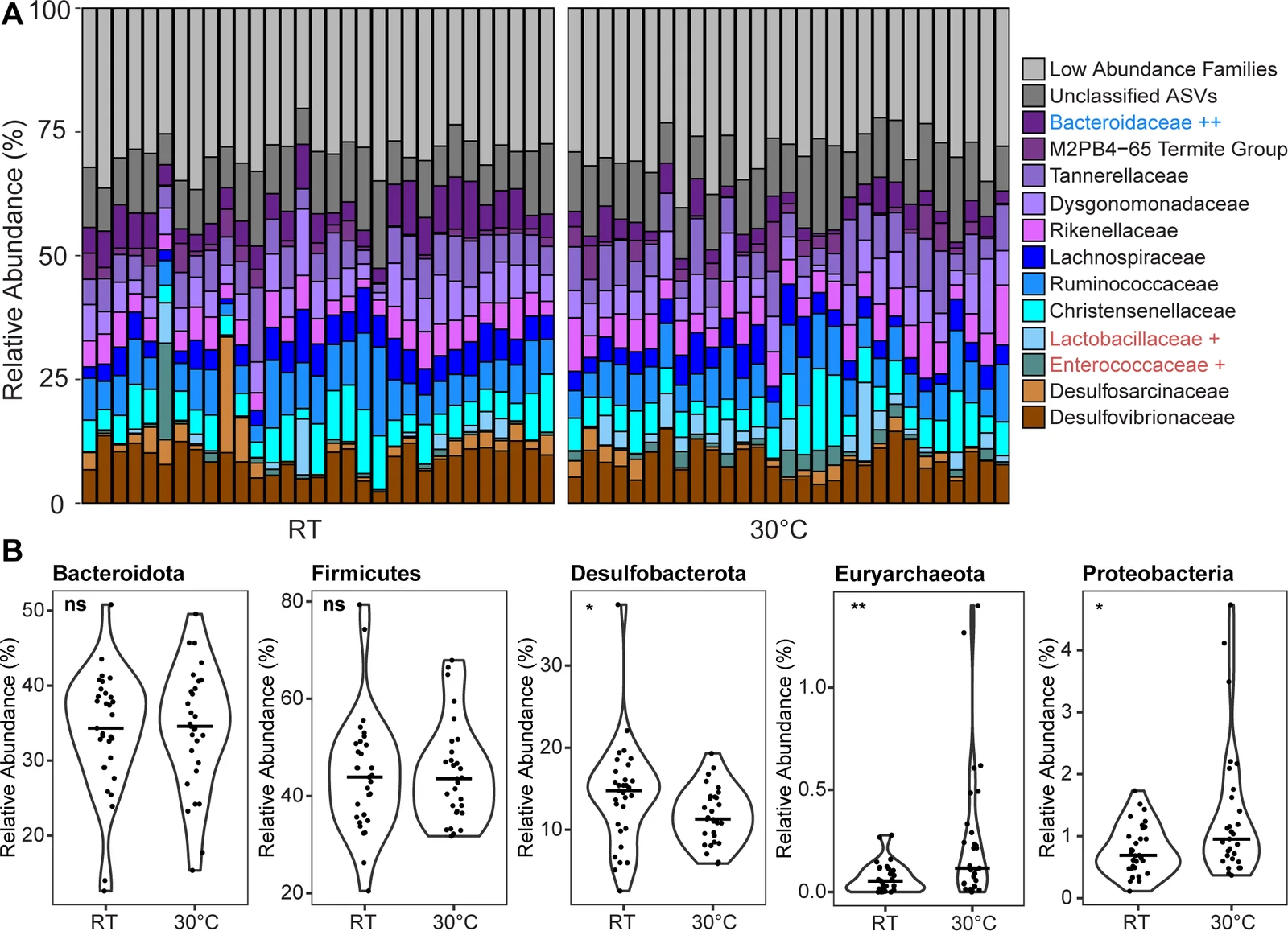
Comparison of family and phylum level relative abundances across temperature treatments. (**A**) Relative abundance of the 12 most abundant families (maximum relative abundance >10%). Each bar represents an individual sample. ++ = higher in room temperature treatment, + = higher in 30°C treatment. (**B**) Violin plots showing the relative abundance of select phyla, where bars represent the median and points represent individual samples. All libraries were resampled to a depth of 10,797 reads. Wilcoxon rank-sum tests were used to compare relative abundance across temperature treatments. ns, no significance; RT, room temperature, *P < 0.05, **P < 0.01.

These ASV level differences translated into significant differences in family and phylum level abundances. At the family level, Wilcoxon rank-sum tests found that Bacteroidaceae were significantly more abundant at room temperature (median difference = 2.07%, *P* < 0.001), while Lactobacillaceae and Enterococcaceae were significantly more abundant at 30°C (median difference = 1.94%, *P* = 0.01 and median difference = 0.41%, *P* = 0.005) (Fig. 5A; Fig. S8). When summed at the phylum level, Bacteroidota and Firmicute (Bacillota) abundances were not significantly different across temperature treatments (*P* = 0.83 and *P* = 0.89). In contrast, the sulfate-reducing Desulfobacterota (Thermodesulfobacteriota) were significantly more abundant at room temperature (median difference = 3.5%, *P* = 0.04), while the Euryarchaeota (containing exclusively *Methanibrevibacter* ASVs) were significantly more abundant at 30°C (median difference = 0.17%, *P* = 0.005). Proteobacteria were significantly more abundant in the 30°C treatment (median difference = 0.26%, *P* = 0.04) (Fig. 5B; Fig. S9).

## DISCUSSION

The goal of this study was to examine how changes in environmental temperature affect gut microbial community composition and stability in omnivorous cockroaches. We observed modest differences between room temperature (20–22°C) and 30°C treatments at the phylum and family level (Fig. 5; Fig. S8 and S9). At the phylum level, there were significantly more Proteobacteria in the 30°C treatment than in the room temperature treatment. The observed shift in Proteobacteria relative abundance was subtle, with a median difference of 0.26% (Fig. 5). This response parallels studies in other insects and invertebrates. In a study of rough wood lice (*Porcellio scaber*), those maintained at 22°C had higher abundances of Proteobacteria (Enterobacteriaceae) compared to those maintained at 15°C (78). Experimental warming of the invertebrate nematode *Caenorhabditis elegans* also resulted in higher abundances of Proteobacteria (*Agrobacterium*) as temperature increased (15°C, 20°C, and 25°C treatments) (79). However, those studies observed more pronounced changes in Proteobacteria abundances compared to our findings in the American cockroach and were concurrent with substantial decreases in other common gut microbiota such as the Actinobacteria in *P. scaber* and *Sphingobacterium* in *C. elegans*. In the closely related termite *Reticulitermes flavipes*, Arango et al. saw a considerable increase in the abundance of Proteobacteria (*Acinetobacter,* Betaproteobacteriales, and *Enterobacter*) at their lowest temperature treatment of 15°C compared to 27°C or 35°C, as well as subsequent decreases in Bacteroidota abundances (51). Studies in the fruit fly *Drosophila melanogaster* and cricket *Gryllus veletus* also report that Proteobacteria are responsive to temperature (80, 81). However, the responsive Proteobacteria in the fruit fly and cricket studies are primarily *Wolbachia*, an intracellular bacterial symbiont that is not directly associated with the gut microbiome.

In vertebrate studies, increases in Proteobacteria abundance are associated with gut dysbiosis, pronounced shifts in community composition, and diseases like irritable bowel syndrome and Crohn’s disease (82–84). The Proteobacteria are generally composed of opportunists that can survive a broad range of stressors compared to other phyla. It may be that changes in their abundance are indirectly determined by other community members’ response to temperature. In this scenario, Proteobacteria are well suited to “take over” under conditions where more typical symbiotic populations are under environmental stress. We observed similar patterns with the Enterococcaceae and Lactobacillaceae in the 30°C treatment (Fig. 4 and 5; Fig. S8), both of which are also commonly associated with gut dysbiosis (85, 86). These taxa appear to bloom in a subset of our 30°C samples, consistent with our observation of increased beta-dispersion under high temperature conditions. This aligns with the “Anna Karenina” model where dysbiotic microbiota are unique and highly variable compared to healthy microbiota (87, 88). We note a similar pattern in *Cephalotes rohweri* ant colonies exposed to high temperatures in field warming studies (27).

We also observed a significant decrease in Desulfobacterota at higher temperatures (Fig. 5). The Desulfobacterota are highly active in *P. americana*, primarily acting as sulfate reducers (34, 89). As sulfate reducers, they are likely in competition with methanogens for hydrogen (90, 91). Cockroach gut methanogenic taxa include the Euryarchaeota, which were significantly more abundant in the 30°C treatment (Fig. 5). This suggests that hydrogen utilization may tip in favor of methanogens at higher temperatures, or that methanogens themselves fare better at higher temperatures, increasing hydrogen utilization and decreasing Desulfobacterota abundances. These observations align with studies in soils and batch reactors, where methanogens outcompete sulfate reducers as temperatures rise (92–94). Further studies are needed to better quantify the methanogenic archaea, as universal 16S rRNA primers used in this study are not optimized for their detection.

The modest shifts in gut microbiome composition that we report here contrast with the experimental warming of *R. flavipes* termites, in which a shift from 27°C to 35°C resulted in large changes in phylum-level composition. Arango et al. reported that Bacteroidetes (Bacteroidota) almost doubled in relative abundance, while Spirochaetes, Euryarchaeota, and Elusimicrobia decreased. However, the authors noted that many of the microbial taxa lost following warming tended to be associated with ciliated protists that do not fare well at these higher temperatures (51). These protists are critical for the breakdown of lignocellulosic material in lower termites (95). While similar protists have been reported in the cockroach, they are not essential for cockroach survival and energy acquisition (96). Microscopic observation of samples from our stock tanks indicates that a few protists are present, but they tend to be present at low abundance and low frequency (unpublished observations). It may be that the “high” temperature tested here was not high enough to negatively impact the protists, or that any reduction in protists had a negligible impact on overall community composition.

Gut microbiome resilience to temperature shifts appears to be unique to cockroaches compared to other insect studies to date. However, comparable trends have been reported in wild *Anolis* lizards, where the gut microbiome was far less responsive to warming environmental temperature than in other lizards (97). While the 30°C temperature used in our study is within the preferred temperature range of this species (53) and well below their upper thermal limit of 39°C (98), it is common for bacteria to have lower tolerances for higher-than-preferred than lower-than-preferred temperatures (99). We also observed that all cockroaches maintained at this temperature had a darker cuticle by the end of the experiment. Cuticle melanization has been directly linked to desiccation tolerance in *Blattella germanica* (100), and desiccation is reported to occur in *P. americana* at 30°C (8.6% average loss of weight per day in dry conditions) (53). Though we do not track desiccation rate or humidity, our observations suggest that the warming treatment was high enough to elicit a melanization stress response in *P. americana*. It is therefore possible that temperature-dependent changes in humidity played a role in the observed responses, likely as a result of changes in desiccation stress.

Together, these results suggest that the cockroach gut microbiome is relatively stable across a wide range of environmental temperatures. The high temperature tested shows some signs of increased variability and growth of opportunistic Proteobacteria, Enterococcaceae, and Lactobacillaceae, which may have implications for the ability of these insects to serve as disease vectors in less climate-controlled settings, for example, increased mechanical transfer frequency of opportunistic pathogens in shared anthropogenic environments. Finally, we highlight the potential for temperature to impact microbial competition between essential hydrogenotrophic organisms in the gut. This study demonstrates that the microbiome of ectotherms can be surprisingly resilient to temperature shifts of up to 10°C and has important implications for the ability of cockroaches to tolerate the impacts of climate change.

Certain limitations of this study merit attention. Host physiological outcomes were not considered, making links between temperature, the gut microbiome, and host fitness not possible. Additionally, our experimental design accounted for adult cockroaches at a single 14-day time point with no recovery period. It is possible that juveniles may respond differently than adults, longer exposure times might elicit more substantial changes to the gut microbiome, and/or the observed changes may be transient. Future studies should consider these factors in their experimental design.

## Data Availability

The sequences generated from this experiment were submitted to the NCBI Sequence Read Archive and are available under BioProject number PRJNA1380472.

## SUPPLEMENTAL MATERIAL

Supplemental figures spectrum.00288-26-s0001.pdf Fig. S1 to S9.

Table S1 spectrum.00288-26-s0002.xlsx Sample metadata and 16S rRNA amplicon read tracking data.

Table S2 spectrum.00288-26-s0003.xlsx Bacterial load qPCR copy data and DNA extraction data.

Table S3 spectrum.00288-26-s0004.xlsx DESeq2 results.

ASM does not own the copyrights to Supplemental Material that may be linked to, or accessed through, an article. The authors have granted ASM a non-exclusive, world-wide license to publish the Supplemental Material files. Please contact the corresponding author directly for reuse.

## References

[1] Stork NE. 2018. How many species of insects and other terrestrial arthropods are there on earth? Annu Rev Entomol 63:31–45. doi:10.1146/annurev-ento-020117-043348 28938083

[2] Whiles MR, Callaham, Jr MA, Meyer CK, Brock BL, Charlton RE. 2001. Emergence of periodical cicadas (*Magicicada cassini)* from a Kansas riparian forest: densities, biomass and nitrogen flux. Am Midl Nat 145:176–187. doi:10.1674/0003-0031(2001)145[0176:EOPCMC]2.0.CO;2

[3] Metcalfe DB, Asner GP, Martin RE, Silva Espejo JE, Huasco WH, Farfán Amézquita FF, Carranza-Jimenez L, Galiano Cabrera DF, Baca LD, Sinca F, Huaraca Quispe LP, Taype IA, Mora LE, Dávila AR, Solórzano MM, Puma Vilca BL, Laupa Román JM, Guerra Bustios PC, Revilla NS, Tupayachi R, Girardin CAJ, Doughty CE, Malhi Y. 2014. Herbivory makes major contributions to ecosystem carbon and nutrient cycling in tropical forests. Ecol Lett 17:324–332. doi:10.1111/ele.12233 24372865

[4] Emerson PM, Lindsay SW, Walraven GEL, Faal H, Bøgh C, Lowe K, Bailey RL. 1999. Effect of fly control on trachoma and diarrhoea. Lancet 353:1401–1403. doi:10.1016/S0140-6736(98)09158-2 10227221

[5] Effler PV, Pang L, Kitsutani P, Vorndam V, Nakata M, Ayers T, Elm J, Tom T, Reiter P, Rigau-Perez JG, Hayes JM, Mills K, Napier M, Clark GG, Gubler DJ, Hawaii Dengue Outbreak Investigation Team. 2005. Dengue fever, Hawaii, 2001–2002. Emerg Infect Dis 11:742–749. doi:10.3201/eid1105.041063 15890132 PMC3320380

[6] Rader R, Bartomeus I, Garibaldi LA, Garratt MPD, Howlett BG, Winfree R, Cunningham SA, Mayfield MM, Arthur AD, Andersson GKS, et al.. 2016. Non-bee insects are important contributors to global crop pollination. Proc Natl Acad Sci USA 113:146–151. doi:10.1073/pnas.1517092112 26621730 PMC4711867

[7] Bowling RD, Brewer MJ, Kerns DL, Gordy J, Seiter N, Elliott NE, Buntin GD, Way MO, Royer TA, Biles S, Maxson E. 2016. Sugarcane aphid (Hemiptera: Aphididae): a new pest on sorghum in North America. J Integr Pest Manag 7:12. doi:10.1093/jipm/pmw011 28446991 PMC5394564

[8] González-Tokman D, Córdoba-Aguilar A, Dáttilo W, Lira-Noriega A, Sánchez-Guillén RA, Villalobos F. 2020. Insect responses to heat: physiological mechanisms, evolution and ecological implications in a warming world. Biol Rev Camb Philos Soc 95:802–821. doi:10.1111/brv.12588 32035015

[9] Del Toro I, Ribbons RR, Ellison AM. 2015. Ant-mediated ecosystem functions on a warmer planet: effects on soil movement, decomposition and nutrient cycling. J Anim Ecol 84:1233–1241. doi:10.1111/1365-2656.12367 25773283

[10] Luo B, Huang M, Wang W, Niu J, Shrestha M, Zeng H, Ma L, Degen AA, Liao J, Zhang T, Bai Y, Zhao J, Fraser LH, Shang Z. 2023. Ant nests increase litter decomposition to mitigate the negative effect of warming in an alpine grassland ecosystem. Proc Biol Sci 290:20230613. doi:10.1098/rspb.2023.0613 37369352 PMC10299860

[11] Diana L, Kevin W. Global temperature - earth indicator. NASA Science. Available from: https://science.nasa.gov/earth/explore/earth-indicators/global-temperature. Accessed 25 March 2026

[12] Lee H, Romero J. 2023. Climate change 2023: synthesis report. Contribution of working groups I, II and III to the sixth assessment report of the intergovernmental panel on climate change. Geneva, Switzerland. IPCC.

[13] Atkinson D. 1994. Temperature and organism size—a biological law for ectotherms? Adv Ecol Res 25:1–58. doi:10.1016/S0065-2504(08)60212-3

[14] Mas‐Martí E, Muñoz I, Oliva F, Canhoto C. 2015. Effects of increased water temperature on leaf litter quality and detritivore performance: a whole-reach manipulative experiment. Freshw Biol 60:184–197. doi:10.1111/fwb.12485

[15] Garrad R, Booth DT, Furlong MJ. 2016. The effect of rearing temperature on development, body size, energetics and fecundity of the diamondback moth. Bull Entomol Res 106:175–181. doi:10.1017/S000748531500098X 26696587

[16] Cummings JH, Pomare EW, Branch WJ, Naylor CPE, Macfarlane GT. 1987. Short chain fatty acids in human large intestine, portal, hepatic and venous blood. Gut 28:1221–1227. doi:10.1136/gut.28.10.1221 3678950 PMC1433442

[17] Ridaura VK, Faith JJ, Rey FE, Cheng J, Duncan AE, Kau AL, Griffin NW, Lombard V, Henrissat B, Bain JR, Muehlbauer MJ, Ilkayeva O, Semenkovich CF, Funai K, Hayashi DK, Lyle BJ, Martini MC, Ursell LK, Clemente JC, Van Treuren W, Walters WA, Knight R, Newgard CB, Heath AC, Gordon JI. 2013. Gut microbiota from twins discordant for obesity modulate metabolism in mice. Science 341:1241214. doi:10.1126/science.1241214 24009397 PMC3829625

[18] Kenny DJ, Plichta DR, Shungin D, Koppel N, Hall AB, Fu B, Vasan RS, Shaw SY, Vlamakis H, Balskus EP, Xavier RJ. 2020. Cholesterol metabolism by uncultured human gut bacteria influences host cholesterol level. Cell Host Microbe 28:245–257. doi:10.1016/j.chom.2020.05.013 32544460 PMC7435688

[19] Reinhardt C, Bergentall M, Greiner TU, Schaffner F, Ostergren-Lundén G, Petersen LC, Ruf W, Bäckhed F. 2012. Tissue factor and PAR1 promote microbiota-induced intestinal vascular remodelling. Nature 483:627–631. doi:10.1038/nature10893 22407318 PMC3885420

[20] Gordon HA, Bruckner-Kardoss E. 1961. Effect of normal microbial flora on intestinal surface area. Am J Physiol-Leg Content 201:175–178. doi:10.1152/ajplegacy.1961.201.1.175 13707165

[21] Banasaz M, Norin E, Holma R, Midtvedt T. 2002. Increased enterocyte production in gnotobiotic rats mono-associated with *Lactobacillus rhamnosus* GG. Appl Environ Microbiol 68:3031–3034. doi:10.1128/AEM.68.6.3031-3034.2002 12039764 PMC123962

[22] Bouskra D, Brézillon C, Bérard M, Werts C, Varona R, Boneca IG, Eberl G. 2008. Lymphoid tissue genesis induced by commensals through NOD1 regulates intestinal homeostasis. Nature 456:507–510. doi:10.1038/nature07450 18987631

[23] Sanos SL, Bui VL, Mortha A, Oberle K, Heners C, Johner C, Diefenbach A. 2009. RORγt and commensal microflora are required for the differentiation of mucosal interleukin 22–producing NKp46^+^ cells. Nat Immunol 10:83–91. doi:10.1038/ni.1684 19029903 PMC4217274

[24] Cash HL, Whitham CV, Behrendt CL, Hooper LV. 2006. Symbiotic bacteria direct expression of an intestinal bactericidal lectin. Science 313:1126–1130. doi:10.1126/science.1127119 16931762 PMC2716667

[25] Prado SS, Hung KY, Daugherty MP, Almeida RPP. 2010. Indirect effects of temperature on stink bug fitness, via maintenance of gut-associated symbionts. Appl Environ Microbiol 76:1261–1266. doi:10.1128/AEM.02034-09 20023083 PMC2820946

[26] Kikuchi Y, Tada A, Musolin DL, Hari N, Hosokawa T, Fujisaki K, Fukatsu T. 2016. Collapse of insect gut symbiosis under simulated climate change. mBio 7:e01578-16. doi:10.1128/mBio.01578-16 27703075 PMC5050343

[27] McMunn MS, Hudson AI, Zemenick AT, Egerer M, Bennett L, Philpott SM, Vannette RL. 2022. Thermal sensitivity and seasonal change in the gut microbiome of a desert ant, *Cephalotes rohweri*. FEMS Microbiol Ecol 98:1–11. doi:10.1093/femsec/fiac062 35641145

[28] Ramsby BD, Hoogenboom MO, Whalan S, Webster NS. 2018. Elevated seawater temperature disrupts the microbiome of an ecologically important bioeroding sponge. Mol Ecol 27:2124–2137. doi:10.1111/mec.14544 29473977

[29] Jokiel PL, Coles SL. 1977. Effects of temperature on the mortality and growth of Hawaiian reef corals. Mar Biol 43:201–208. doi:10.1007/BF00402312

[30] Li Y-F, Yang N, Liang X, Yoshida A, Osatomi K, Power D, Batista FM, Yang J-L. 2018. Elevated seawater temperatures decrease microbial diversity in the gut of *Mytilus coruscus*. Front Physiol 9:839. doi:10.3389/fphys.2018.00839 30042689 PMC6049046

[31] Renelies-Hamilton J, Germer K, Sillam-Dussès D, Bodawatta KH, Poulsen M. 2021. Disentangling the relative roles of vertical transmission, subsequent colonizations, and diet on cockroach microbiome assembly. mSphere 6:e01023-20. doi:10.1128/mSphere.01023-20 33408228 PMC7845597

[32] Cazzaniga M, Domínguez-Santos R, Marín-Miret J, Gil R, Latorre A, García-Ferris C. 2023. Exploring gut microbial dynamics and symbiotic interaction in *Blattella germanica* using rifampicin. Biology (Basel) 12:955. doi:10.3390/biology12070955 37508385 PMC10376618

[33] Tinker KA, Ottesen EA. 2021. Differences in gut microbiome composition between sympatric wild and allopatric laboratory populations of omnivorous cockroaches. Front Microbiol 12:703785. doi:10.3389/fmicb.2021.703785 34394050 PMC8355983

[34] Dukes HE, Tinker KA, Ottesen EA. 2023. Disentangling hindgut metabolism in the American cockroach through single-cell genomics and metatranscriptomics. Front Microbiol 14:1156809. doi:10.3389/fmicb.2023.1156809 37323917 PMC10266427

[35] Dockman RL, Ottesen EA. 2024. Purified fibers in chemically defined synthetic diets destabilize the gut microbiome of an omnivorous insect model. Front Microbiomes 3:1477521. doi:10.3389/frmbi.2024.1477521 40114931 PMC11925550

[36] Jahnes BC, Poudel K, Staats AM, Sabree ZL. 2021. Microbial colonization promotes model cockroach gut tissue growth and development. J Insect Physiol 133:104274. doi:10.1016/j.jinsphys.2021.104274 34216600

[37] Jahnes BC, Herrmann M, Sabree ZL. 2019. Conspecific coprophagy stimulates normal development in a germ-free model invertebrate. PeerJ 7:e6914. doi:10.7717/peerj.6914 31139506 PMC6521811

[38] Turner M, Van Hulzen L, Pietri JE. 2023. The gut microbiota induces melanin deposits that act as substrates for *fimA*-mediated aggregation of *Salmonella* Typhimurium and enhance infection of the German cockroach vector. Microbiol Spectr 11:e0211923. doi:10.1128/spectrum.02119-23 37606369 PMC10580948

[39] Turner M, Van Hulzen L, Guse K, Agany D, Pietri JE. 2024. The gut microbiota confers resistance against *Salmonella* Typhimurium in cockroaches by modulating innate immunity. iScience 27:111293. doi:10.1016/j.isci.2024.111293 39628558 PMC11612784

[40] Wada-Katsumata A, Zurek L, Nalyanya G, Roelofs WL, Zhang A, Schal C. 2015. Gut bacteria mediate aggregation in the German cockroach. Proc Natl Acad Sci USA 112:15678–15683. doi:10.1073/pnas.1504031112 26644557 PMC4697420

[41] Tinker KA, Ottesen EA. 2020. Phylosymbiosis across deeply diverging lineages of omnivorous cockroaches (order Blattodea). Appl Environ Microbiol 86:e02513-19. doi:10.1128/AEM.02513-19 31953337 PMC7082566

[42] Irmler L, Furch K. 1979. Production, energy, and nutrient turnover of the cockroach Epilampra irmleri Rocha e Silva & Aguiar in a Central-Amazonian inundation forest. Amazoniana 6:497–520.

[43] Wang C, Lee C-Y, Rust MK. 2021. Biology and management of the German cockroach. CSIRO Publishing, Clayton South VIC, Australia.

[44] Arbes Jr. SJ, Gergen PJ, Elliott L, Zeldin DC. 2005. Prevalences of positive skin test responses to 10 common allergens in the US population: results from the Third National Health and Nutrition Examination Survey. J Allergy Clin Immunol 116:377–383. doi:10.1016/j.jaci.2005.05.017 16083793

[45] Kang B, Vellody D, Homburger H, Yunginger JW. 1979. Cockroach cause of allergic asthma. Its specificity and immunologic profile. J Allergy Clin Immunol 63:80–86. doi:10.1016/0091-6749(79)90196-9 83332

[46] Memona H, Manzoor F, Anjum AA. 2017. Cockroaches (Blattodea: Blattidae): a reservoir of pathogenic microbes in human-dwelling localities in Lahore. J Med Entomol 54:435–440. doi:10.1093/jme/tjw168 27744361

[47] Liu J, Yuan Y, Feng L, Lin C, Ye C, Liu J, Li H, Hao L, Liu H. 2024. Intestinal pathogens detected in cockroach species within different food-related environment in Pudong, China. Sci Rep 14:1947. doi:10.1038/s41598-024-52306-x 38253647 PMC10803747

[48] Rosenstreich DL, Eggleston P, Kattan M, Baker D, Slavin RG, Gergen P, Mitchell H, McNiff-Mortimer K, Lynn H, Ownby D, Malveaux F. 1997. The role of cockroach allergy and exposure to cockroach allergen in causing morbidity among inner-city children with asthma. N Engl J Med 336:1356–1363. doi:10.1056/NEJM199705083361904 9134876

[49] Gulati J. 2025. Pest control in the US. 56171. IBIS World Industry Report

[50] Inward D, Beccaloni G, Eggleton P. 2007. Death of an order: a comprehensive molecular phylogenetic study confirms that termites are eusocial cockroaches. Biol Lett 3:331–335. doi:10.1098/rsbl.2007.0102 17412673 PMC2464702

[51] Arango RA, Schoville SD, Currie CR, Carlos-Shanley C. 2021. Experimental warming reduces survival, cold tolerance, and gut prokaryotic diversity of the eastern subterranean termite, *Reticulitermes flavipes* (Kollar). Front Microbiol 12:632715. doi:10.3389/fmicb.2021.632715 34079527 PMC8166220

[52] Arango RA, Bishell AB, Ohno KM, Shelton TG, Schoville SD, Carlos-Shanley C. 2024. Seasonal shifts in gut microbiota and cold tolerance metrics in a northern population of *Reticulitermes flavipes* (Blattodea: Rhinotermitidae). Environ Entomol 53:447–456. doi:10.1093/ee/nvae027 38574195

[53] Gunn DL. 1935. The temperature and humidity relations of the cockroach: III. A comparison of temperature preference, and rates of desiccation and respiration of *Periplaneta americana*, *Blatta orientalis* and *Blatella germanica*. J Exp Biol 12:185–190. doi:10.1242/jeb.12.2.185

[54] Ayayee PA, Ondrejech A, Keeney G, Muñoz-Garcia A. 2018. The role of gut microbiota in the regulation of standard metabolic rate in female *Periplaneta americana*. PeerJ 6:e4717. doi:10.7717/peerj.4717 29844953 PMC5971104

[55] Lee S, Kim JY, Yi M, Lee I-Y, Lee W-J, Moon HS, Yong D, Yong T-S. 2020. Comparative microbiome analysis of three species of laboratory-reared *Periplaneta* cockroaches. Korean J Parasitol 58:537–542. doi:10.3347/kjp.2020.58.5.537 33202505 PMC7672242

[56] Vera-Ponce de León A, Jahnes BC, Otero-Bravo A, Sabree ZL. 2021. Microbiota perturbation or elimination can inhibit normal development and elicit a starvation-like response in an omnivorous model invertebrate. mSystems 6:e0080221. doi:10.1128/mSystems.00802-21 34427529 PMC8407121

[57] Chen Z, Wen S, Shen J, Wang J, Liu W, Jin X. 2023. Composition and diversity of the gut microbiota across different life stages of American cockroach (*Periplaneta americana*). Bull Entomol Res 113:787–793. doi:10.1017/S0007485323000469 38037350

[58] Bertino-Grimaldi D, Medeiros MN, Vieira RP, Cardoso AM, Turque AS, Silveira CB, Albano RM, Bressan-Nascimento S, Garcia ES, de Souza W, Martins OB, Machado EA. 2013. Bacterial community composition shifts in the gut of *Periplaneta americana* fed on different lignocellulosic materials. Springerplus 2:609. doi:10.1186/2193-1801-2-609 24324923 PMC3855920

[59] Zurek L, Keddie BA. 1996. Contribution of the colon and colonie bacterial flora to metabolism and development of the american cockroach *Periplaneta americana* L. J Insect Physiol 42:743–748. doi:10.1016/0022-1910(96)00028-5

[60] Rollo CD, Gunderman MW. 1984. Variation among individuals and the effect of temperature on food consumption and reproduction in the cockroach, *Periplaneta americana* (Orthoptera: Blattidae). Can Entomol 116:785–793. doi:10.4039/Ent116785-6

[61] Nadkarni MA, Martin FE, Jacques NA, Hunter N. 2002. Determination of bacterial load by real-time PCR using a broad-range (universal) probe and primers set. Microbiology (Reading, Engl) 148:257–266. doi:10.1099/00221287-148-1-257 11782518

[62] Wickham H, Averick M, Bryan J, Chang W, McGowan LD, François R, Grolemund G, Hayes A, Henry L, Hester J, Kuhn M, Pedersen TL, Miller E, Bache SM, Müller K, Ooms J, Robinson D, Seidel DP, Spinu V, Takahashi K, Vaughan D, Wilke C, Woo K, Yutani H. 2019. Welcome to the Tidyverse. J Open Source Softw 4:1686. doi:10.21105/joss.01686

[63] Tinker KA, Ottesen EA. 2016. The core gut microbiome of the American cockroach, *Periplaneta americana*, is stable and resilient to dietary shifts. Appl Environ Microbiol 82:6603–6610. doi:10.1128/AEM.01837-16 27590811 PMC5086554

[64] Callahan BJ, McMurdie PJ, Rosen MJ, Han AW, Johnson AJA, Holmes SP. 2016. DADA2: high-resolution sample inference from Illumina amplicon data. Nat Methods 13:581–583. doi:10.1038/nmeth.3869 27214047 PMC4927377

[65] Quast C, Pruesse E, Yilmaz P, Gerken J, Schweer T, Yarza P, Peplies J, Glöckner FO. 2013. The SILVA ribosomal RNA gene database project: improved data processing and web-based tools. Nucleic Acids Res 41:D590–D596. doi:10.1093/nar/gks1219 23193283 PMC3531112

[66] R Core Team. 2022. R: a language and environment for statistical computing. Vienna, Austria. R Foundation for Statistical Computing.

[67] Oksanen J, Simpson GL, Blanchet FG, Kindt R, Legendre P, Minchin PR, O’Hara RB, Solymos P, Stevens MHH, Szoecs E, et al.. 2022. vegan: community ecology package (2.6-4).

[68] Legendre P, De Cáceres M. 2013. Beta diversity as the variance of community data: dissimilarity coefficients and partitioning. Ecol Lett 16:951–963. doi:10.1111/ele.12141 23809147

[69] Ling W, Lu J, Zhao N, Lulla A, Plantinga AM, Fu W, Zhang A, Liu H, Song H, Li Z, Chen J, Randolph TW, Koay WLA, White JR, Launer LJ, Fodor AA, Meyer KA, Wu MC. 2022. Batch effects removal for microbiome data via conditional quantile regression. Nat Commun 13:5418. doi:10.1038/s41467-022-33071-9 36109499 PMC9477887

[70] Love MI, Huber W, Anders S. 2014. Moderated estimation of fold change and dispersion for RNA-seq data with DESeq2. Genome Biol 15:550. doi:10.1186/s13059-014-0550-8 25516281 PMC4302049

[71] Sievers F, Wilm A, Dineen D, Gibson TJ, Karplus K, Li W, Lopez R, McWilliam H, Remmert M, Söding J, Thompson JD, Higgins DG. 2011. Fast, scalable generation of high‐quality protein multiple sequence alignments using Clustal Omega. Mol Syst Biol 7:539. doi:10.1038/msb.2011.75 21988835 PMC3261699

[72] Price MN, Dehal PS, Arkin AP. 2009. FastTree: computing large minimum evolution trees with profiles instead of a distance matrix. Mol Biol Evol 26:1641–1650. doi:10.1093/molbev/msp077 19377059 PMC2693737

[73] Price MN, Dehal PS, Arkin AP. 2010. FastTree 2 – approximately maximum-likelihood trees for large alignments. PLoS One 5:e9490. doi:10.1371/journal.pone.0009490 20224823 PMC2835736

[74] Yu G, Smith DK, Zhu H, Guan Y, Lam T-Y. 2017. ggtree: an r package for visualization and annotation of phylogenetic trees with their covariates and other associated data. Methods Ecol Evol 8:28–36. doi:10.1111/2041-210X.12628

[75] Gu Z. 2022. Complex heatmap visualization. iMeta 1:e43. doi:10.1002/imt2.43 38868715 PMC10989952

[76] Gu Z, Eils R, Schlesner M. 2016. Complex heatmaps reveal patterns and correlations in multidimensional genomic data. Bioinformatics 32:2847–2849. doi:10.1093/bioinformatics/btw313 27207943

[77] Ogle DH, Doll JC, Wheeler AP, Dinno A. 2025. FSA: simple fisheries stock assessment methods (0.9.6)

[78] Horváthová T, Babik W, Kozłowski J, Bauchinger U. 2019. Vanishing benefits - The loss of actinobacterial symbionts at elevated temperatures. J Therm Biol 82:222–228. doi:10.1016/j.jtherbio.2019.04.015 31128651

[79] Berg M, Stenuit B, Ho J, Wang A, Parke C, Knight M, Alvarez-Cohen L, Shapira M. 2016. Assembly of the *Caenorhabditis elegans* gut microbiota from diverse soil microbial environments. ISME J 10:1998–2009. doi:10.1038/ismej.2015.253 26800234 PMC5029150

[80] Ferguson LV, Dhakal P, Lebenzon JE, Heinrichs DE, Bucking C, Sinclair BJ. 2018. Seasonal shifts in the insect gut microbiome are concurrent with changes in cold tolerance and immunity. Funct Ecol 32:2357–2368. doi:10.1111/1365-2435.13153

[81] Moghadam NN, Thorshauge PM, Kristensen TN, de Jonge N, Bahrndorff S, Kjeldal H, Nielsen JL. 2018. Strong responses of *Drosophila melanogaster* microbiota to developmental temperature. Fly (Austin) 12:1–12. doi:10.1080/19336934.2017.1394558 29095113 PMC5927714

[82] Willing B, Halfvarson J, Dicksved J, Rosenquist M, Järnerot G, Engstrand L, Tysk C, Jansson JK. 2009. Twin studies reveal specific imbalances in the mucosa-associated microbiota of patients with ileal Crohn’s disease. Inflamm Bowel Dis 15:653–660. doi:10.1002/ibd.20783 19023901

[83] Willing BP, Dicksved J, Halfvarson J, Andersson AF, Lucio M, Zheng Z, Järnerot G, Tysk C, Jansson JK, Engstrand L. 2010. A pyrosequencing study in twins shows that gastrointestinal microbial profiles vary with inflammatory bowel disease phenotypes. Gastroenterology 139:1844–1854. doi:10.1053/j.gastro.2010.08.049 20816835

[84] Su Q, Tun HM, Liu Q, Yeoh YK, Mak JWY, Chan FKL, Ng SC. 2023. Gut microbiome signatures reflect different subtypes of irritable bowel syndrome. Gut Microbes 15:2157697. doi:10.1080/19490976.2022.2157697 36573834 PMC9809927

[85] Dahal RH, Kim S, Kim YK, Kim ES, Kim J. 2023. Insight into gut dysbiosis of patients with inflammatory bowel disease and ischemic colitis. Front Microbiol 14:1174832. doi:10.3389/fmicb.2023.1174832 37250025 PMC10211348

[86] Pietrucci D, Cerroni R, Unida V, Farcomeni A, Pierantozzi M, Mercuri NB, Biocca S, Stefani A, Desideri A. 2019. Dysbiosis of gut microbiota in a selected population of Parkinson’s patients. Parkinsonism Relat Disord 65:124–130. doi:10.1016/j.parkreldis.2019.06.003 31174953

[87] Zaneveld JR, McMinds R, Vega Thurber R. 2017. Stress and stability: applying the Anna Karenina principle to animal microbiomes. Nat Microbiol 2:17121. doi:10.1038/nmicrobiol.2017.121 28836573

[88] Ma ZS. 2020. Testing the Anna Karenina principle in human microbiome-associated diseases. iScience 23:101007. doi:10.1016/j.isci.2020.101007 32305861 PMC7163324

[89] DePoy AN, Wall HE, Tinker KA, Black IM, Ottesen EA. 2025. Metabolic flexibility among fiber degraders supports gut microbiome stability across host diets in the American cockroach. bioRxiv. doi:10.1101/2024.10.31.621369

[90] Gibson GR, Cummings JH, Macfarlane GT. 1988. Competition for hydrogen between sulphate-reducing bacteria and methanogenic bacteria from the human large intestine. J Appl Bacteriol 65:241–247. doi:10.1111/j.1365-2672.1988.tb01891.x 2852666

[91] Lovley DR, Dwyer DF, Klug MJ. 1982. Kinetic analysis of competition between sulfate reducers and methanogens for hydrogen in sediments. Appl Environ Microbiol 43:1373–1379. doi:10.1128/aem.43.6.1373-1379.1982 16346033 PMC244242

[92] Bodegom P van, Stams AJM. 1999. Effects of alternative electron acceptors and temperature on methanogenesis in rice paddy soils. Chemosphere 39:167–182. doi:10.1016/S0045-6535(99)00101-0

[93] Muller E, Guélard J, Sissmann O, Tafit A, Poirier S. 2024. Evidencing the influence of temperature and mineralogy on microbial competition for hydrogen consumption: implications for underground hydrogen storage (UHS). Int J Hydrogen Energy 82:1101–1113. doi:10.1016/j.ijhydene.2024.08.024

[94] Jung H, Kim J, Lee C. 2019. Temperature effects on methanogenesis and sulfidogenesis during anaerobic digestion of sulfur-rich macroalgal biomass in sequencing batch reactors. Microorganisms 7:682. doi:10.3390/microorganisms7120682 31835811 PMC6955875

[95] Todaka N, Moriya S, Saita K, Hondo T, Kiuchi I, Takasu H, Ohkuma M, Piero C, Hayashizaki Y, Kudo T. 2007. Environmental cDNA analysis of the genes involved in lignocellulose digestion in the symbiotic protist community of *Reticulitermes speratus*. FEMS Microbiol Ecol 59:592–599. doi:10.1111/j.1574-6941.2006.00237.x 17239084

[96] Gijzen HJ, Barugahare M. 1992. Contribution of anaerobic protozoa and methanogens to hindgut metabolic activities of the American cockroach, *Periplaneta americana*. Appl Environ Microbiol 58:2565–2570. doi:10.1128/aem.58.8.2565-2570.1992 1514803 PMC195822

[97] Williams CE, Kueneman JG, Nicholson DJ, Rosso AA, Folfas E, Casement B, Gallegos-Koyner MA, Neel LK, Curlis JD, McMillan WO, Cox CL, Logan ML. 2022. Sustained drought, but not short-term warming, alters the gut microbiomes of wild *Anolis* lizards. Appl Environ Microbiol 88:e0053022. doi:10.1128/aem.00530-22 36165625 PMC9552597

[98] Gunn DL, Notley FB. 1936. The temperature and humidity relations of the cockroach. IV. Thermal death-point. J Exp Biol 13:28–34. doi:10.1242/jeb.13.1.28

[99] Bennett AF, Lenski RE. 1993. Evolutionary adaptation to temperature II. Thermal niches of experimental lines of *Escherichia coli*. Evolution (N Y) 47:1–12. doi:10.1111/j.1558-5646.1993.tb01194.x 28568084

[100] Bai T-T, Pei X-J, Liu T-X, Fan Y-L, Zhang S-Z. 2022. Melanin synthesis genes BgTH and BgDdc affect body color and cuticle permeability in *Blattella germanica*. Insect Sci 29:1552–1568. doi:10.1111/1744-7917.13024 35191584

