# Supplementary material for: Large temperature excursions have modest impacts on community composition in the high diversity gut microbiome of omnivorous American cockroaches (Periplaneta americana)
Source: Microbiol Spectr. 2026 Jun 1;14(7):e00288-26. doi: 10.1128/spectrum.00288-26 (PMC13340255; doi:10.1128/spectrum.00288-26)
Supplement: Supplemental figures — Fig. S1 to S9. [file spectrum.00288-26-s0001.pdf]

# Supplementary Material

## Large temperature excursions have modest impacts on community composition in the high diversity gut microbiome of omnivorous American cockroaches (*Periplaneta americana)*

**Kevin C. Riedmuller[a], Josey E. Dyer[a]*, Elizabeth A. Ottesen[a]#**

## Contents

**Description of supplementary tables** (uploaded as separate files) 2

**Fig S1:** Comparison of between cohort Bray-Curtis dissimilarities 3

**Fig S2:** Family level comparison of hindgut microbial community composition across temperature treatments 4

**Fig S3:** Comparison of hindgut microbial community composition between male and female cockroaches 5

**Fig S4:** Evaluation of temperature effects on the microbial community composition of male cockroaches. 6

**Fig S5:** Evaluation of temperature effects on the microbial community composition of female cockroaches. 7

**Fig S6:** Heatmap of differentially abundant ASVs across temperature treatments 8

**Fig S7:** Relative abundance of the most differentially abundant ASVs across temperature treatments 9

**Fig S8:** Family level relative abundances across temperature treatments 10

**Fig S9:** Phylum level relative abundances across temperature treatments 11

## Description of supplementary tables

**Table S1:** Sample metadata and 16S rRNA amplicon read tracking data.

**Table S2:** Bacterial load qPCR copy data and DNA extraction data.

**Table S3:** DESeq2 results table. Sheet one contains all results. The remaining sheets are split by the most abundant families (maximum relative abundance greater than 10%) and ASVs are filtered for significance ($p < 0.05$).

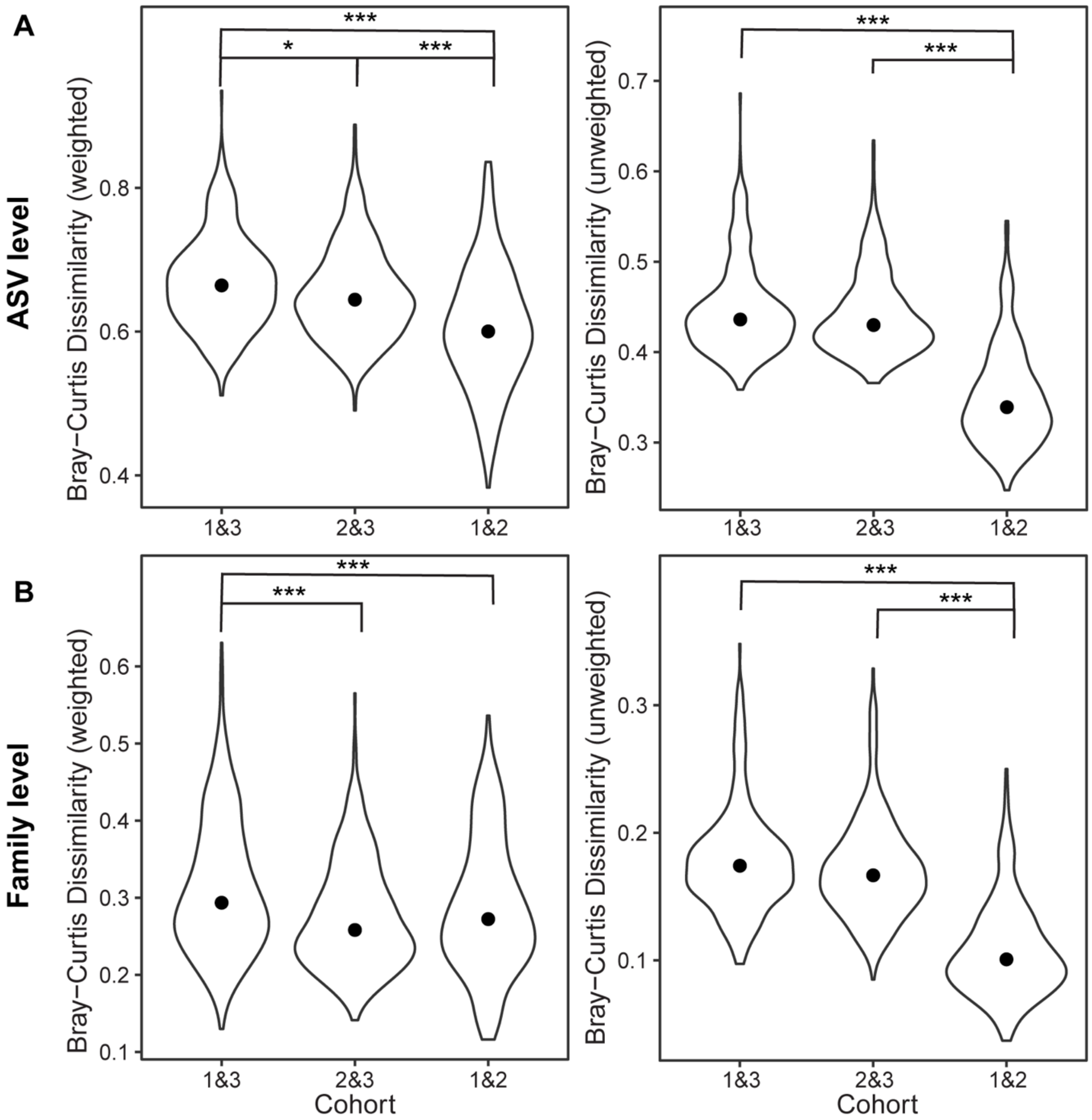


**Fig S1. Comparison of between cohort Bray-Curtis dissimilarities.**

Violin plots of weighted (left) and unweighted (right) Bray-Curtis dissimilarities between cohorts at the (A) ASV and (B) family level where points represent the medians. Kruskal-Wallis and post-hoc Dunn's test with Bonferroni adjustment were used to compare groups. All libraries were resampled to a depth of 10,797 reads. * = $p < 0.05$, *** = $p < 0.001$.

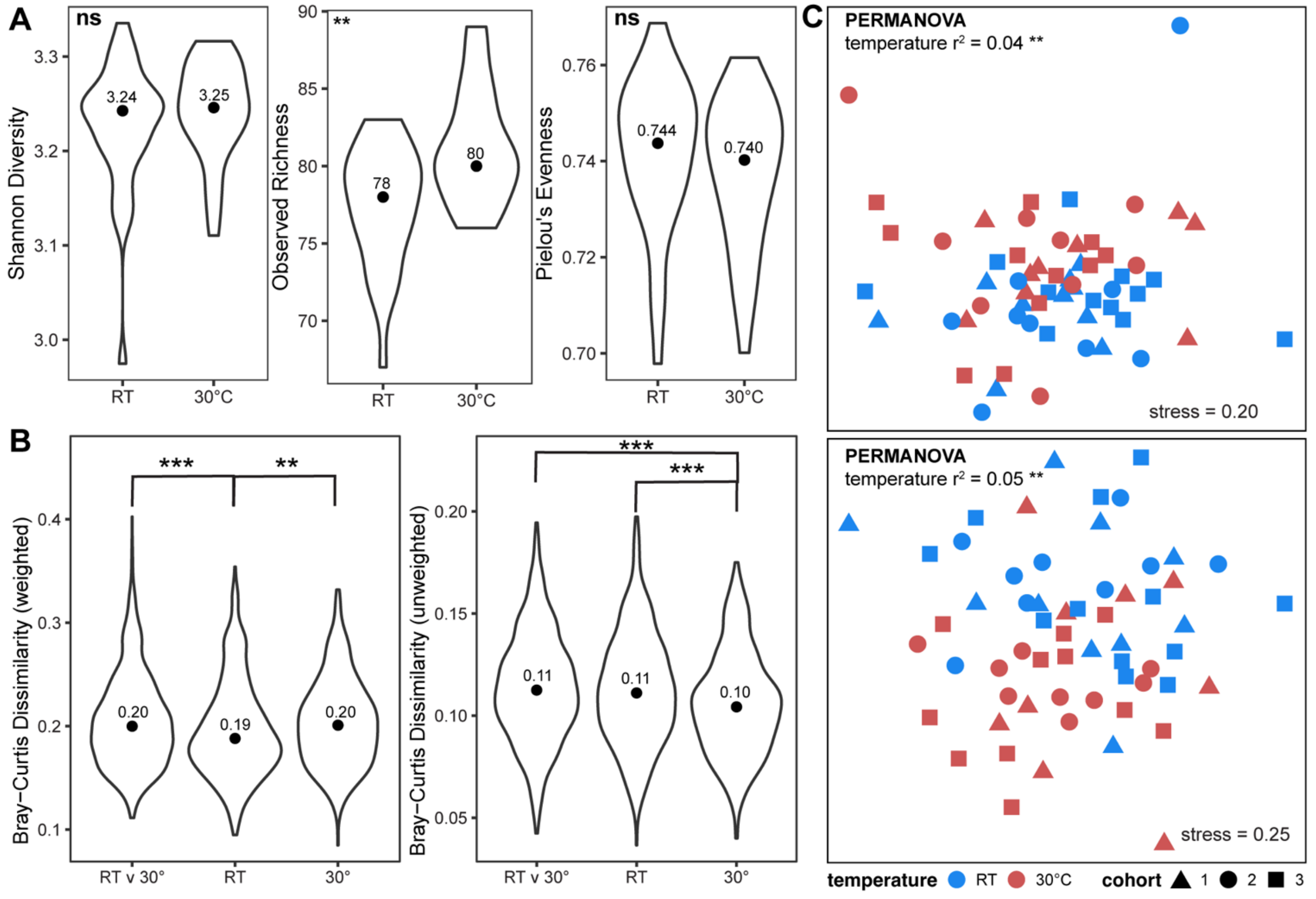


**Fig S2. Family level comparison of hindgut microbial community composition across temperature treatments.**

(A) Violin plots of alpha diversity measurements (Shannon diversity, observed richness, Pielou's evenness) where points represent the medians. (B) Violin plots of weighted (left) and unweighted (right) Bray-Curtis dissimilarities within and between temperature treatments where points represent the medians. Wilcoxon rank-sum tests were used to compare alpha diversity measures. Kruskal-Wallis and post-hoc Dunn's test with Bonferroni adjustment were used to compare Bray-Curtis dissimilarities. (C) Nonmetric multidimensional scaling (NMDS) of weighted (top) and unweighted (bottom) Bray-Curtis dissimilarities. NMDS stress was calculated with the metaMDS() function from the Vegan package. PERMANOVA was used to calculate $r^2$ and $p$ values. All libraries were batch corrected and resampled to a depth of 10,098 reads. RT = room temperature, ** = $p < 0.01$, *** = $p < 0.001$, ns = no significance.

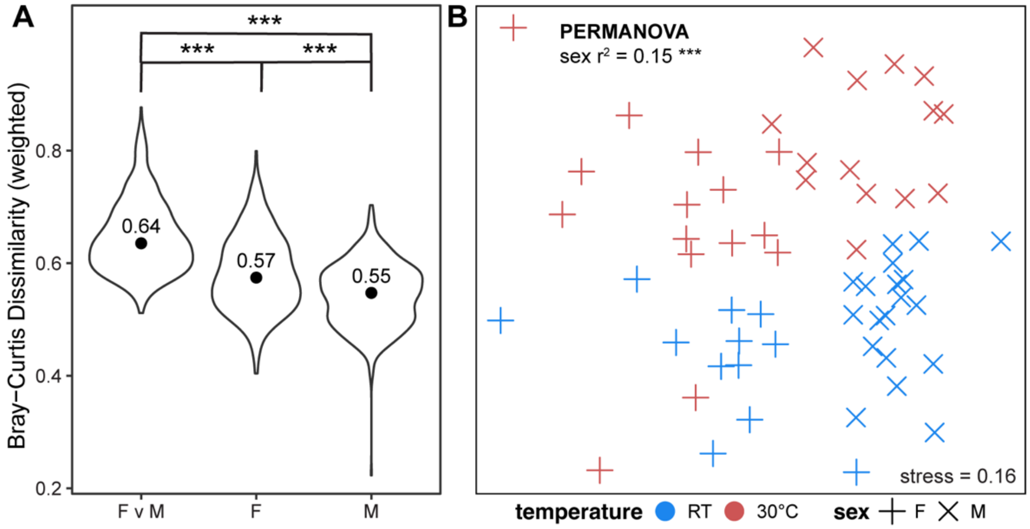


**Fig S3. Comparison of hindgut microbial community composition between male and female cockroaches.** (A) Violin plots of weighted Bray-Curtis dissimilarities within and between male and female cockroaches where points represent the medians. Kruskal-Wallis and post-hoc Dunn's test with Bonferroni adjustment were used to compare Bray-Curtis dissimilarities. (B) Nonmetric multidimensional scaling (NMDS) of weighted Bray-Curtis dissimilarities. NMDS stress was calculated with the metaMDS() function from the Vegan package. PERMANOVA was used to calculate $r^2$ and $p$ values. All libraries were batch corrected and resampled to a depth of 10,098 reads. RT = room temperature, *** = $p < 0.001$.

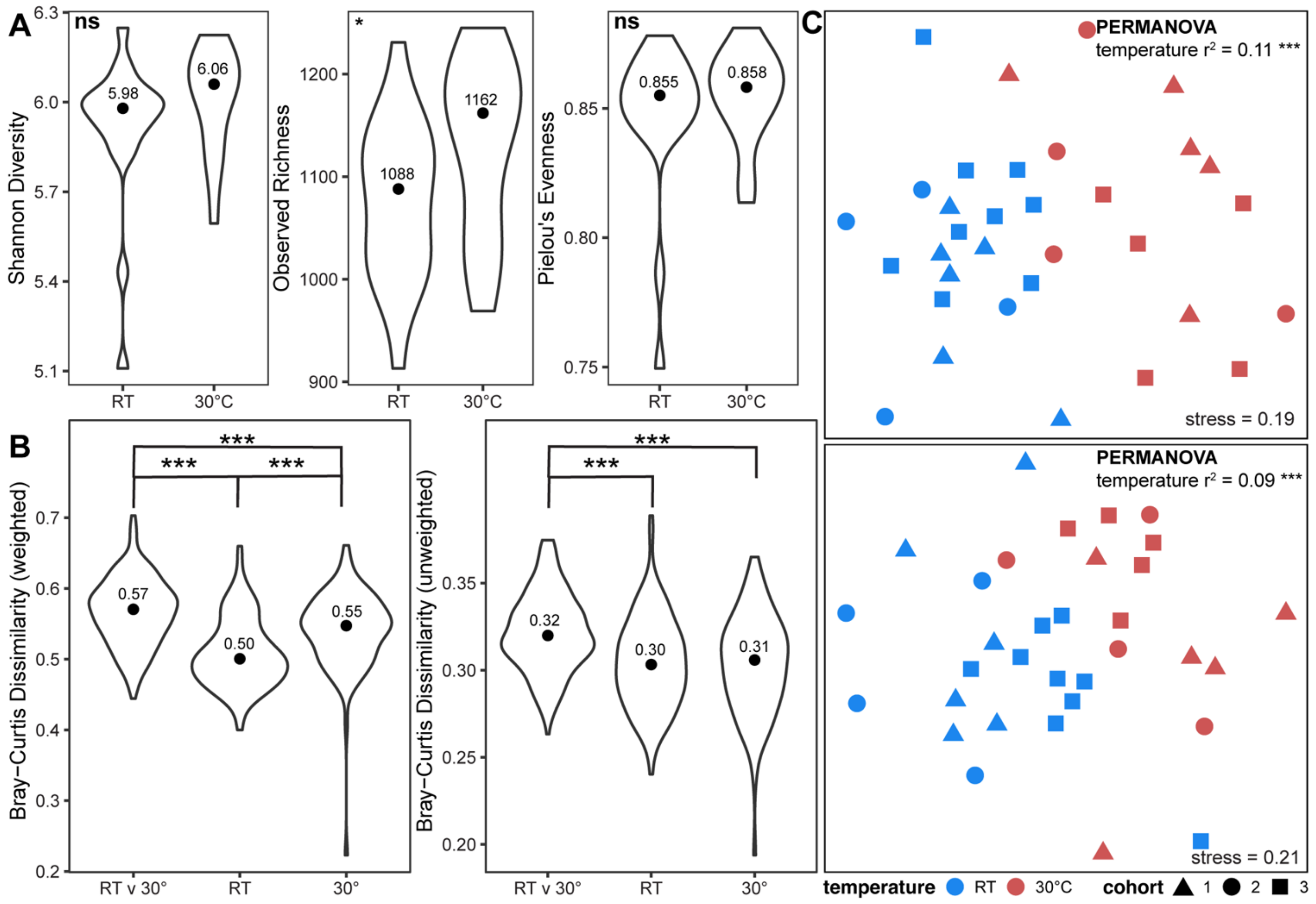


**Fig S4. Evaluation of temperature effects on the microbial community composition of male cockroaches.** (A) Violin plots of alpha diversity measurements (Shannon diversity, observed richness, Pielou's evenness) where points represent the medians. (B) Violin plots of weighted (left) and unweighted (right) Bray-Curtis dissimilarities within and between temperature treatments where points represent the medians. Wilcoxon rank-sum tests were used to compare alpha diversity measures. Kruskal-Wallis and post-hoc Dunn's test with Bonferroni adjustment were used to compare Bray-Curtis dissimilarities. (C) Nonmetric multidimensional scaling (NMDS) of weighted (top) and unweighted (bottom) Bray-Curtis dissimilarities. NMDS stress was calculated with the metaMDS() function from the Vegan package. PERMANOVA was used to calculate $r^2$ and $p$ values. All libraries were batch corrected and resampled to a depth of 10,098 reads. RT = room temperature, * = $p < 0.05$, *** = $p < 0.001$, ns = no significance.

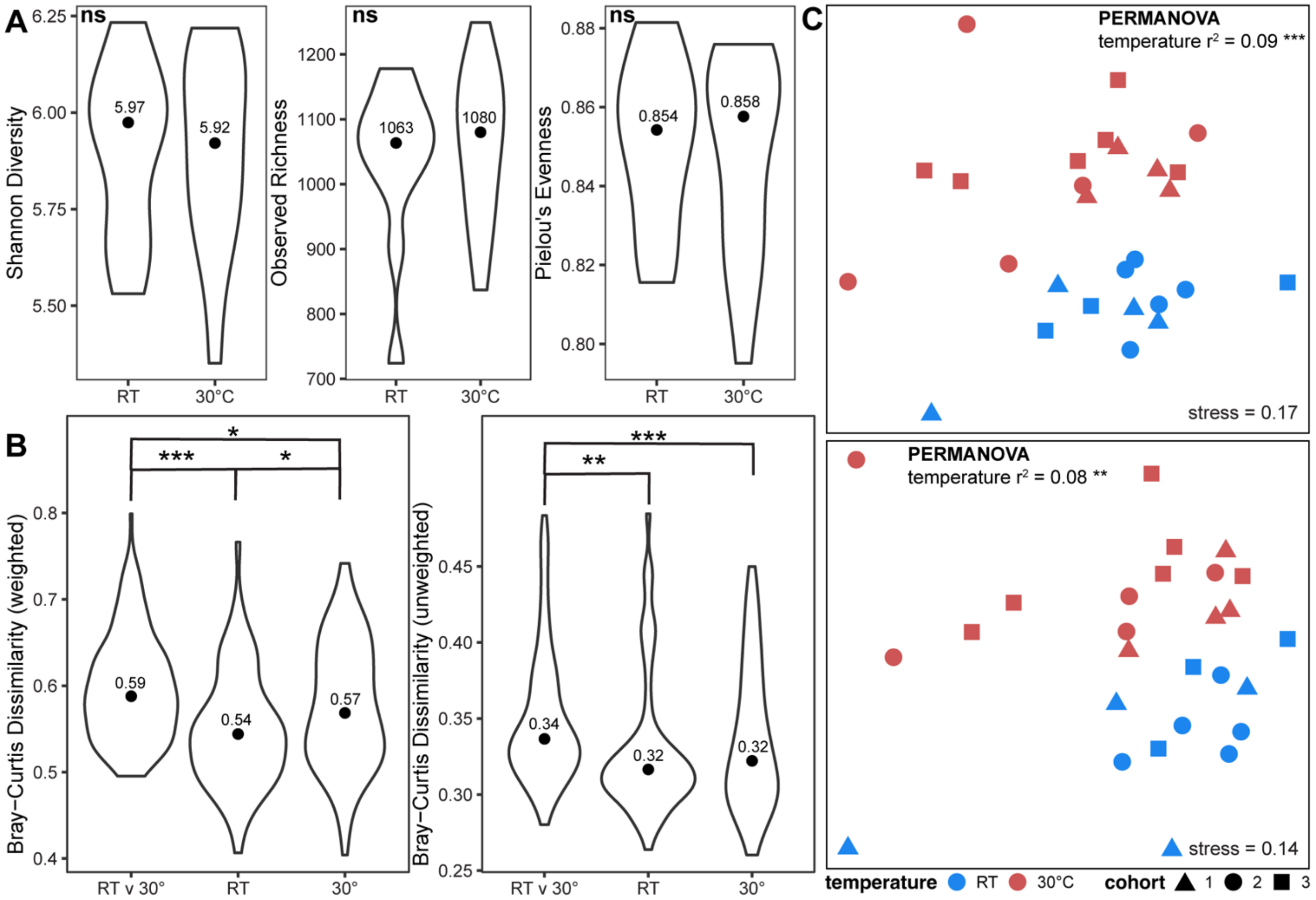


**Fig S5. Evaluation of temperature effects on the microbial community composition of female cockroaches.**

(A) Violin plots of alpha diversity measurements (Shannon diversity, observed richness, Pielou's evenness) where points represent the medians. (B) Violin plots of weighted (left) and unweighted (right) Bray-Curtis dissimilarities within and between temperature treatments where points represent the medians. Wilcoxon rank-sum tests were used to compare alpha diversity measures. Kruskal-Wallis and post-hoc Dunn's test with Bonferroni adjustment were used to compare Bray-Curtis dissimilarities. (C) Nonmetric multidimensional scaling (NMDS) of weighted (top) and unweighted (bottom) Bray-Curtis dissimilarities. NMDS stress was calculated with the metaMDS() function from the Vegan package. PERMANOVA was used to calculate $r^2$ and $p$ values. All libraries were batch corrected and resampled to a depth of 10,098 reads. RT = room temperature, * = $p < 0.05$, ** = $p < 0.01$, *** = $p < 0.001$, ns = no significance.

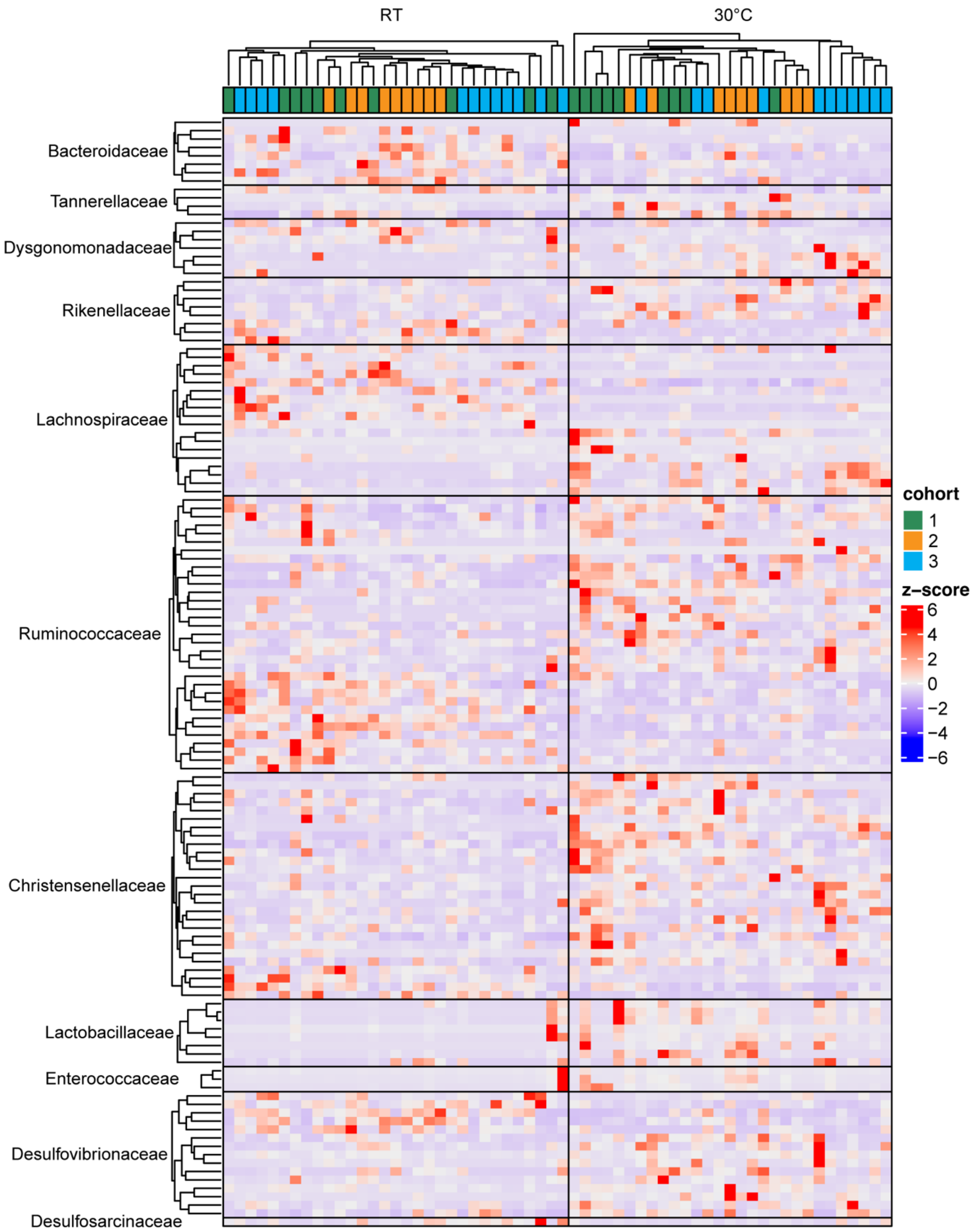


**Fig S6: Heatmap of differentially abundant ASVs across temperature treatments.**

Heatmap depicting the relative abundance of differentially abundant ASVs in the most abundant families (maximum relative abundance > 10%) as determined by DESeq2 (Table S3) ($p < 0.05$). Heatmap was generated using the ComplexHeatmap package with default clustering parameters. Relative abundances were Z-score standardized by row. RT = room temperature.

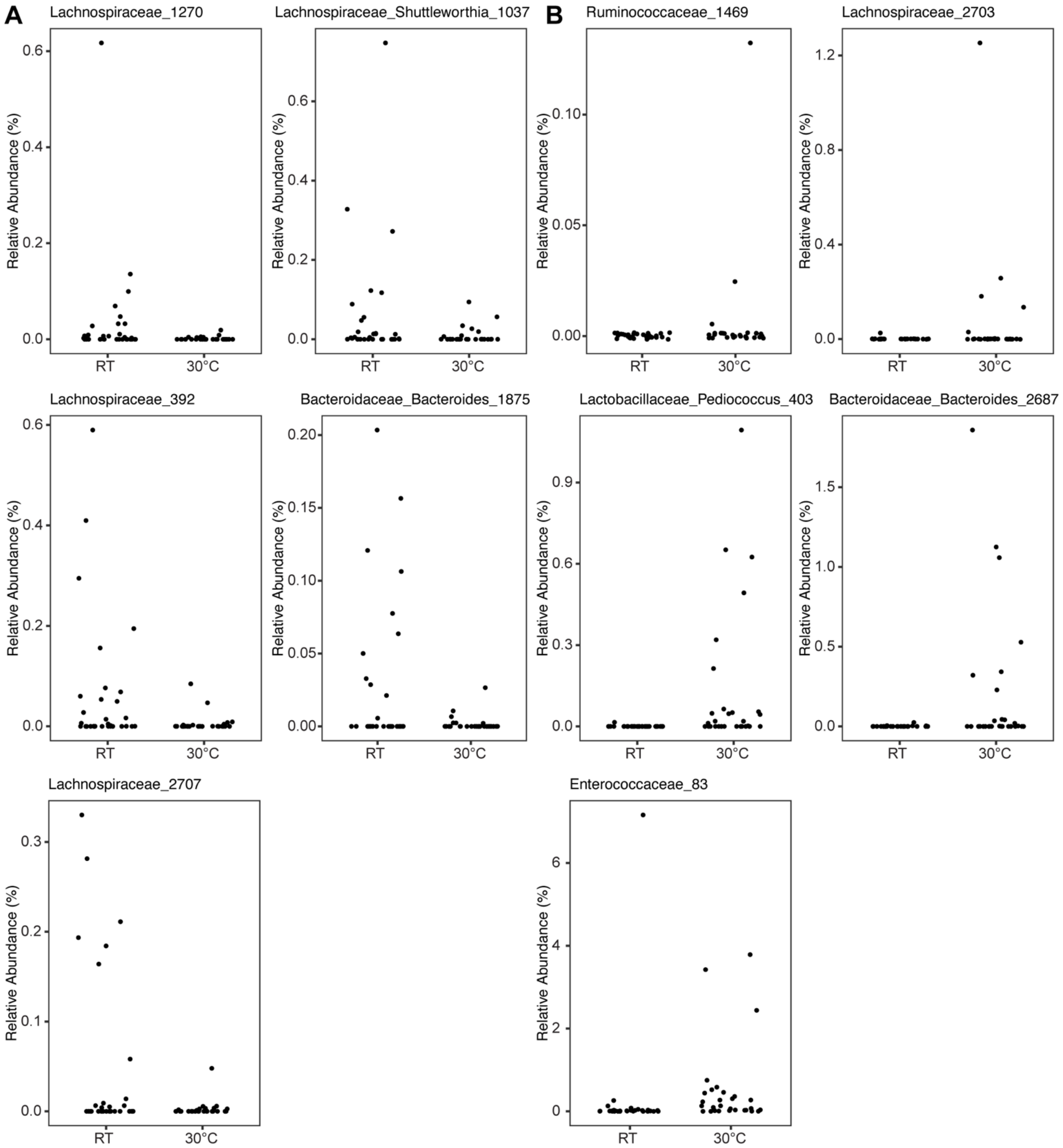


**Fig S7. Relative abundance of the most differentially abundant ASVs across temperature treatments.** Jitter plots illustrating the relative abundance of ASVs with the largest log2 fold change in either direction as determined by DESeq2. ASVs were selected from the most abundant families (maximum relative abundance > 10%). (A) highlights 5 ASVs with the most negative log2 fold change (higher in RT) and (B) highlights 5 ASVs with the greatest positive log2 fold change (higher in 30°C). RT = room temperature.

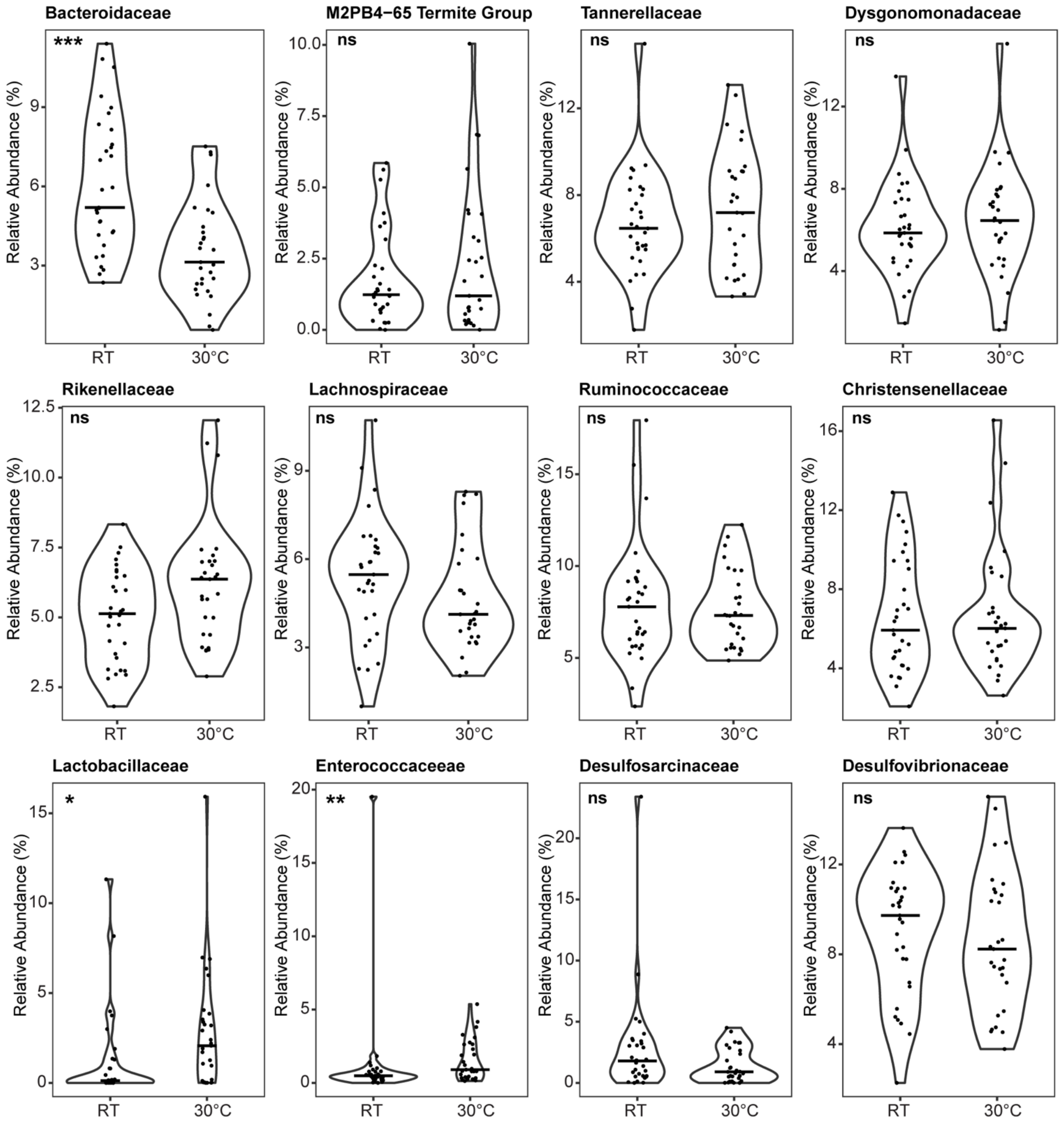


**Fig S8. Family level relative abundances across temperature treatments.**

Violin plots showing the relative abundances of the 12 most abundant families (maximum relative abundance > 10%). Bars represent the median and points represent individual samples. Wilcoxon rank-sum tests were used to compare relative abundances across temperature treatments. RT = room temperature, * = $p < 0.05$, ** = $p < 0.01$, *** = $p < 0.001$, ns = no significance.

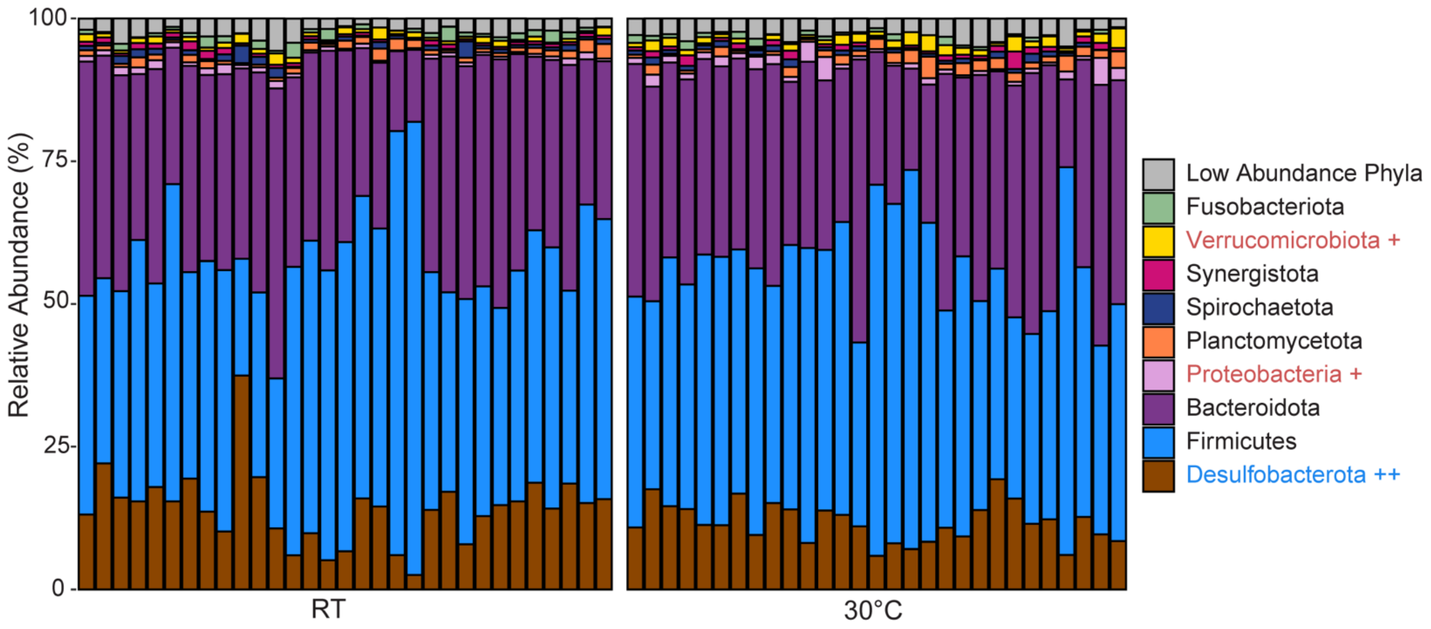


**Fig S9. Phylum level relative abundances across temperature treatments.**

Relative abundances of the 9 most abundant phyla (maximum relative abundance > 2%). Each stacked bar represents an individual sample. ++ = higher in room temperature treatment, + = higher in 30°C treatment as determined by Wilcoxon rank-sum tests ($p < 0.05$). RT = room temperature.
